# Characterization of the Genetic Diversity of Extensively-Drug Resistant *Mycobacterium tuberculosis* Clinical Isolates from Pulmonary Tuberculosis Patients in Peru

**DOI:** 10.1371/journal.pone.0112789

**Published:** 2014-12-09

**Authors:** Omar Cáceres, Nalin Rastogi, Carlos Bartra, David Couvin, Marco Galarza, Luis Asencios, Alberto Mendoza-Ticona

**Affiliations:** 1 Biotechnology and Molecular Biology Laboratory, Instituto Nacional de Salud, Lima, Peru; 2 WHO Supranational TB Reference Laboratory, TB and Mycobacteria Unit, Institut Pasteur de la Guadeloupe, Guadeloupe, France; 3 Mycobacterias National Reference Laboratory, Instituto Nacional de Salud, Lima, Peru; 4 Ministerio de Salud (MINSA), Lima, Peru; University of Padova, Medical School, Italy

## Abstract

**Background:**

Peru holds the fourth highest burden of tuberculosis in the Americas. Despite an apparently well-functioning DOTS control program, the prevalence of multidrug resistant tuberculosis (MDR-TB) continues to increase. To worsen this situation, cases of extensively drug resistance tuberculosis (XDR-TB) have been detected. Little information exists about the genetic diversity of drug-susceptible vs. MDR-TB and XDR-TB.

**Methods:**

Cryopreserved samples of XDR strains from 2007 to 2009 (second semester), were identified and collected. Starting from 227 frozen samples, a total of 142 XDR-TB strains of *Mycobacterium tuberculosis* complex (MTBC; 1 isolate per patient) were retained for this study. Each strain DNA was analyzed by spoligotyping and the 15-loci Mycobacterial Interspersed Repetitive Unit (MIRU-15).

**Results:**

Among the 142 isolates analyzed, only 2 samples (1.41%) could not be matched to any lineage. The most prevalent sublineage was Haarlem (43.66%), followed by T (27.46%), LAM (16.2%), Beijing (9.15%), and X clade (1.41%). Spoligotype analysis identified clustering for 128/142 (90.1%) isolates vs. 49/142 (34.5%) with MIRUs. Of the samples, 90.85% belonged to retreated patients. The drug resistant profile demonstrated that 62.67% showed resistance to injectable drugs capreomycin (CAP) and kanamycin (KAN) vs. 15.5% to CAP alone and 21.8% to KAN alone. The SIT219/T1 and SIT50/H3 were the most prevalent patterns in our study. The spoligoforest analysis showed that SIT53/T1 was at the origin of many of the T lineage strains as well as a big proportion of Haarlem lineage strains (SIT50/H3, followed by SIT47/H1, SIT49/H3, and SIT2375/H1), as opposed to the SIT1/Beijing strains that did not appear to evolve into minor Beijing sublineages among the XDR-TB strains.

**Conclusion:**

In contrast with other Latin-American countries where LAM sublineage is the most predominant, we found the Haarlem to be the most common followed by T sublineage among the XDR-TB strains.

## Introduction

With almost 9 million new cases in 2011 and 1.4 million deaths, tuberculosis (TB) caused by *Mycobacterium tuberculosis* ranks as the second leading cause of death from an infectious disease in the world [1]. The emergence of multidrug-resistant (MDR) strains showing combined resistance to two major first-line drugs isoniazid (INH) and rifampicin (RIF) and the increased HIV/TB coinfection not only contribute to the spread and re-emergence of this disease, but also constitute a threat of developing added resistance to second-line drugs.

From 2007 to 2010 the proportion of new TB cases reported as MDR-TB ranged from 0%–28.9% while the proportion of previously treated MDR-TB ranged from 0% to 65.1% [2]. MDR-TB complicates management of patients due to increased pressure on public health systems and cost of the treatment. It further aggravates the emergence of extensively drug-resistant TB (XDR-TB), defined as MDR-TB plus resistance to a fluoroquinolone and at least one of the three second-line injectable drugs (Amikacin, Kanamycin or Capreomycin). The fact that XDR-TB requires longer, more expensive and more toxic treatment regimens, that at the same time are less likely to cure the disease [2], further worsens the situation. Hence, tackling XDR-TB represents a formidable challenge to public health programs, particularly in low-resource settings.

Following Haiti, Bolivia and Guyana, Peru holds the fourth highest burden of tuberculosis in the Americas. In 2012, 29,760 cases were reported with an incidence of 95 cases per 100,000 inhabitants [1]. MDR-TB in Peru is increasing; in 2012, 1225 new cases of pulmonary MDR-TB were reported [1]. Peru has 41.3% of all MDR-TB cases in the region of the Americas.

The first XDR-TB cases were detected by Instituto Nacional de Salud (INS) in 2007 [3]. Since then, the number of new XDR-TB cases has been increasing, e.g., 50 new cases were detected in 2010 vs. 92 in 2013. From the total cases of TB, the highest prevalence of MDR-TB and XDR-TB cases occurred in Lima (the capital of Peru) with 80% and 92% respectively [4].

To better comprehend the molecular epidemiology of MTBC, techniques based on two-step typing strategies combining rapid and high resolution PCR-based methods such as spoligotyping [5] and MIRU-VNTRs [6], [7] have been successfully used. Among the latter, 15-loci MIRU-VNTRs were shown to possess enough discriminatory power for epidemiological studies permitting assignment of clusters with epidemiological data efficiently [7], [8]. We therefore decided to characterize the genetic diversity of the XDR-TB strains isolated from pulmonary TB patients in Peru, and to analyze their population structure using spoligotyping and 15-loci MIRU-VNTRs in conjunction with available demographic, clinical and epidemiological data.

## Materials and Methods

### Bacteria and strain information

The Mycobacteria Laboratory at the INS is the National Reference Laboratory for the diagnosis and detection of MTBC drug resistance in Peru. Under its routine activity, INS isolated, identified, and cryo-preserved all MDR and XDR strains in a MTBC strain bank, which were duly confirmed for their drug-resistance using drug-susceptibility testing (DST) for first and second line antituberculosis drugs by the agar proportion method [9]. Starting from 227 frozen samples obtained between 2007 to the second semester of 2009, a total of 142 XDR-TB strains of *Mycobacterium tuberculosis* complex (MTBC; 1 isolate per patient) were successfully subcultured, and retained for this retrospective genotyping study. All cryo-preserved strains were thawed and reactivated in 2 ml of 7H9 liquid culture media for 10 days. After the strains were confirmed to be MTBC by rapid chromatographic immunoassay (BD MGI TBc Identification Test), 1.5 ml of culture was centrifuged at 10000 rpm for 5 minutes. The pellet was resuspended in 1 ml of TE buffer, 500 uL of the resuspension was heated at 100°C for 30 minutes, and the DNA was extracted using the CTAB-NaCl method [10]. The remaining 0.5 ml was subcultured in Löwenstein-Jensen (L-J) medium to be cryopreserved thereafter.

For strain information, an Excel database was generated with demographic data (age, sex, geographic area of isolation, year of isolation) and drug resistant profiles (RIF: rifampicin, INH: isoniazid, EMB: ethambutol, PZ: pyrazinamide, SM: streptomycin, CFX: ciprofloxacin, KAN: kanamycin, CAP: capreomycin, ETH: ethionamide, PAS: p-amino salicylic acid, CS: cycloserine). All of this data was taken from the lab registry for cryo-preserved strains. Each sample evaluated was a single strain from a unique patient. Although serial strains are available in the strain bank, we were careful to only examine one strain per patient for this analysis. The strains covered all Peruvian departments where XDR strains were primarily isolated.

### Genotyping methods

Spoligotyping was carried out using a commercial kit (Isogen Bioscience, BV Maarsen, The Netherlands) according to the protocol previously described by Kamerbeek et al. [5]. Briefly, the DR region of the TB genome was amplified using primers DRa and DRb, and the amplified biotinylated products were hybridized to a set of 43 oligonucleotides covalently bound to a membrane. The hybridized PCR products were then incubated with a streptavidin-peroxidase conjugate and the membrane then exposed to chemiluminescence reaction (Amersham ECL Direct nucleic acid labeling and detection system, GE Healthcare Limited, UK). The membrane was exposed on a gel documentation system (Chemidoc XRS, Biorad, USA). DNA extracts of *M. tuberculosis* H37Rv and *M. bovis* BCG were used as controls. Spoligotypes in binary format were analyzed and compared with the SITVIT2 proprietary database of the Pasteur Institute of Guadeloupe, which is an updated “in-house” version of the SITVITWEB database [11]. A cluster was defined as two or more strains sharing identical spoligotyping patterns, and assigned a Spoligotype International Type (SIT) number in the database.

15-loci MIRU typing was performed as described elsewhere [8]. Briefly, each locus was amplified individually using 2 µL of mycobacterial DNA (20 ng) in 23 µL of a reaction mixture containing 0.4 µM of loci-respective primers and PCR Master Mix (Invitrogen, California, USA) according to the manufacturer's instructions. The PCR conditions for each set of primers were carried out as described [8] with a minor modification; we used Betaine 1M instead of DMSO. PCR products were subjected to electrophoresis in a 2% weight/volume agarose gel (Invitrogen Life Technologies, SP, Brazil). 100-bp DNA Ladders (Fermentas, Vilnius, Lithuania) were used as molecular markers. The gels were stained with ethidium bromide and visualized under ultraviolet light, then photodocumented with Chemidoc XRS System. PCR fragment size was determined by Quantity One software (BioRad, CA, USA) with the molecular markers as reference and the MIRU allele scoring was determined according to Supply et al. [6]. For data entry in the SITVIT2 database, the results from each of the 15 loci were combined to create a 15-digit allelic profile in the following order: MIRU-4, MIRU-10, MIRU-16, MIRU-26, MIRU-31, MIRU-40, ETR-A, ETR-C, QUB-11b, QUB-26, QUB-4156, Mtub04, Mtub21, Mtub30, and Mtub39. A cluster was defined as two or more strains sharing identical 15-loci MIRU patterns, and assigned a MIRU International Type (15-MIT) number in the database.

### Evolutionary relationship analysis

A minimum spanning tree (MST) illustrating evolutionary relationships between spoligotypes and MIRU patterns was drawn using BioNumerics version 6.6 (Applied Maths NV, Sint-Maartens-Latem, Belgium). MST connects each spoligotype based on the degree of changes required to go from one allele to another. The MST structure is represented by branches (continuous vs. dashed and dotted lines) and circles representing each individual pattern. The length of the branches represents the distance between patterns while the complexity of the lines (continuous, gray dashed and gray dotted) denotes the number of allele/spacer changes between two patterns: solid lines, 1 or 2 or 3 changes (thicker ones indicate a single change, while the thinner ones indicate 2 or 3 changes); gray dashed lines represent 4 changes; and gray dotted lines represent 5 or more changes. The size of the circle is proportional to the total number of isolates.

We drew MSTs to visualize the relationships between spoligotype patterns and the sites of isolation of the strains, the treatment history of patients, and resistance to injectable drugs, namely CAP and KAN. Peruvian XDR strains belonging to Beijing lineage were further compared by drawing a MST with Beijing isolates from other countries in the SITVIT2 database (n = 863); for this analysis we retained countries with a significant number of Beijing strains with 15-loci MIRU typing data (35 or more) being available, i.e., Japan (n = 603), China (n = 209) and France (n = 35).

In addition, the comparative diversity of these strains was also evaluated by WebLogo graphical representation (available at: http://weblogo.berkeley.edu) which was previously used to represent spoligotyping motifs based on the presence or absence of specific spacer sequences [12]. This application was initially designed to generate a graphical representation of amino acid or nucleic acid sequence logo analysis [13], [14]. We adapted this application to create sequence codes for 15-loci MIRUs as follows; WebLogo Label/number of copies for a loci: A/1, B/2, C/3, D/4, E/5, F/6, G/7, H/8, I/9, J/10, K/11, L/12, M/13, N/14, O/15, P/16, Q/0, U/Unknown.

Relationships among spoligotypes were estimated using the spoligoforest program in the SpolTools webpage (http://www.emi.unsw.edu.au/spoltools/; [15], [16]) for all SITs observed. The method makes use of a model that considers mutations in spoligotypes as irreversible deletions of spacers, and assigns probabilities to the lengths of these deletions. The size of each node is an increasing function of the number of isolates (i.e., the cluster size); edges between nodes reflect evolutionary relationships between spoligotypes with arrowheads pointing to descendants. The spoligoforest tree was colored using the GraphViz software (http://www.graphviz.org).

### Genetic diversity analysis

The Hunter–Gaston discriminatory index (HGDI) [17] was used to estimate the discriminatory power of genotyping methods. Cluster analyses of Spoligotyping and MIRU profiles were also recorded as character data and analyzed using MIRU-VNTRplus program [18]. Dendrograms were generated by using the Jaccard's distance option and the unweighted pair group method of averages (UPGMA) clustering method.

### Statistical analysis

Statistical analysis was performed with Epi Info software 3.51 (Centers for Disease Control and Prevention, Atlanta, GA, USA), by using χ2 test or Fisher exact test for the comparison of proportions. Median age and interquartile (IQ) ranges were calculated using MegaStat software (http://highered.mheducation.com/sites/0077425995/information_center_view0/index.html). A p value<0.04 was considered significant, and a p value between 0.04 and 0.06 was considered “marginally significant”.

### Ethical consideration

The study was approved at the Institutional Review Board and Ethical Committee at Instituto Nacional de Salud in Peru. All information related to the patients was completely anonymized prior to analysis.

## Results

### Characteristics of the population studied

Starting from 227 frozen samples in the INS strain-bank, a total of 142 XDR-TB strains of *Mycobacterium tuberculosis* complex (MTBC; 1 isolate per patient) representing 62.5% of the sample were retained for this study; 85 strains were not included in the study for a diversity of reasons (strains not reactivated, duplicated strains from the same patient and/or epidemiological data missing). All subsequent analyses are based on the 142 MTBC strains genotyped under this study. The age of the patients ranged between 15 and 72 years, with an average of 34 years and a median of 31 years (**S1 Table**). The majority of the subjects (66.2%) were aged between 25 and 54 years. From this sample, 90 (63.4%) were male and 52 (36.6%) were female. In regards to age groups the difference between male and female was marginally significant (p = 0.0505) (Table 1).

**Table 1:** Age distribution between males and females with XDR-TB.

Age Groups	Frequency in Females	Percent	Frequency in Males	Percent
1<21	4	7.7	9	10.0
21<41	40	76.9	49	54.4
41<61	7	13.5	27	30.0
61<81	1	1.9	5	5.6
Total	52	100.0	90	100.0

p-value = 0.050579770369946.

The difference between Females and Males is marginally significant (regarding the Age groups).

All samples were pulmonary XDR-TB and HIV negative, 13 samples (9.15%) were new XDR-TB cases and 129 (90.85%) were relapsed. DST confirmed that all samples were XDR-TB, but showed a variable resistance to injectable drugs: 89 samples (62.67%) showed resistance to CAP and KAN, 22 (15.5%) were resistant to CAP alone and 31 samples (21.8%) were resistant to KAN alone. (**S2 Table**)

In regards to the origin of the strains, 119 (83.8%) were isolated in Lima and 23 samples (16.2%) in one of the following departments: Ica, Tacna, Arequipa Madre de Dios, Junin and Callao (constitutional province). The cases detected in Callao represented 60.8% of all cases detected outside of Lima.

### Analysis by spoligotyping

We performed spoligotyping to determine the population structure of the 142 XDR strains (**S2 Table**). The most dominant spoligotype family in the XDR cases was the Haarlem (H) sublineage (43.6%, n = 62), followed by the T (28.2%, n = 40), Latin-American and Mediterranean (LAM, 16.2%, n = 23), Beijing (9.2%, n = 13) and X3 (1.4%, n = 2) sublineages. Two isolates (1.4%) displayed unknown patterns with no matches to any of the major clades present in the database. Moreover, we found nine (6.3%) new SITs (**Table 2**) and 5 orphan strains (3.5%) that were not present in the SITVIT2 database (see **S2 Table** for detailed genotyping and drug-resistance data and demographic information).

**Table 2:** Description of 26 shared-types (SITs; n = 137 isolates) and corresponding spoligotyping defined lineages/sublineages starting from a total of 227 cryopreserved *M. tuberculosis* strains isolated from adults with pulmonary tuberculosis in Lima, Peru.

SIT	Spoligotype Description	Octal Number	Nb in this study	% in study	% in this study vs. database	Lineage**	Match with a strain from (for newly created SITs)***	Unique vs. Clustered¥
1	□□□□□□□□□□□□□□□□□□□□□□□□□□□□□□□□□□▪▪▪▪▪▪▪▪▪	000000000003771	13	9.15	0.13	Beijing		Clustered
42	▪▪▪▪▪▪▪▪▪▪▪▪▪▪▪▪▪▪▪▪□□□□▪▪▪▪▪▪▪▪□□□□▪▪▪▪▪▪▪	777777607760771	4	2.82	0.12	LAM9		Clustered
47	▪▪▪▪▪▪▪▪▪▪▪▪▪▪▪▪▪▪▪▪▪▪▪▪▪□□□□□□▪□□□□▪▪▪▪▪▪▪	777777774020771	16	11.27	1.07	H1		Clustered
49	▪▪▪▪▪▪▪▪▪▪▪▪▪▪▪▪▪▪▪▪▪▪▪▪▪▪▪▪▪▪□▪□□□□▪▪▪□▪▪▪	777777777720731	4	2.82	2.26	H3		Clustered
50	▪▪▪▪▪▪▪▪▪▪▪▪▪▪▪▪▪▪▪▪▪▪▪▪▪▪▪▪▪▪□▪□□□□▪▪▪▪▪▪▪	777777777720771	21	14.79	0.63	H3		Clustered
52	▪▪▪▪▪▪▪▪▪▪▪▪▪▪▪▪▪▪▪▪▪▪▪▪▪▪▪▪▪▪▪▪□□□□▪▪▪□▪▪▪	777777777760731	3	2.11	0.33	T2		Clustered
53	▪▪▪▪▪▪▪▪▪▪▪▪▪▪▪▪▪▪▪▪▪▪▪▪▪▪▪▪▪▪▪▪□□□□▪▪▪▪▪▪▪	777777777760771	7	4.93	0.12	T1		Clustered
91	▪▪▪□□□□□□□□□□▪▪▪▪□▪▪▪▪▪▪▪▪▪▪▪▪▪▪□□□□▪▪▪▪▪▪▪	700036777760771	1	0.7	0.4	X3		Unique
93	▪▪▪▪▪▪▪▪▪▪▪▪□▪▪▪▪▪▪▪□□□□▪▪▪▪▪▪▪▪□□□□▪▪▪▪▪▪▪	777737607760771	3	2.11	0.84	LAM5		Clustered
189	▪▪▪▪▪▪▪▪▪▪▪▪▪□□□□▪▪▪▪▪▪▪▪▪▪▪▪▪▪▪□□□□▪▪▪▪▪▪▪	777741777760771	1	0.7	9.09	T1		Unique
219	▪▪▪▪▪▪▪▪▪▪▪▪▪□□□□□▪▪▪▪▪▪▪▪▪▪▪▪▪▪□□□□▪▪▪▪▪▪▪	777740777760771	21	14.79	35.59	T1		Clustered
222	▪▪▪▪▪▪▪▪▪▪▪▪▪▪▪▪□□□□□▪▪▪▪▪▪▪□▪▪▪□□□□▪▪▪▪▪▪▪	777774077560771	1	0.7	1.89	Unknown		Unique
291	▪▪▪▪▪▪▪▪▪▪▪▪▪▪▪▪▪▪▪▪□▪▪▪▪▪▪▪▪▪▪▪□□□□▪▪▪▪▪▪▪	777777677760771	1	0.7	1.15	T1		Unique
469	□□□▪▪▪▪▪▪▪▪▪▪▪▪▪▪▪▪▪□□□□▪▪▪▪▪▪▪▪□□□□▪▪▪▪▪▪▪	077777607760771	5	3.52	14.71	LAM1		Clustered
1122	▪▪▪▪▪□▪▪▪▪▪▪▪▪▪▪▪▪▪▪▪▪▪▪▪▪▪▪▪▪▪▪□□□□▪▪▪▪▪▪▪	767777777760771	1	0.7	1.69	T1		Unique
1160	▪▪▪▪▪▪▪▪▪▪▪▪□▪▪▪▪▪□▪□□□□▪▪▪▪▪▪▪▪□□□□▪▪▪▪▪▪▪	777737207760771	1	0.7	25	LAM5		Unique
1355	▪▪▪▪▪▪▪▪▪▪▪▪▪▪▪▪▪▪▪□□□□□▪▪▪▪□▪▪▪□□□□▪▪▪□▪▪▪	777777407560731	7	4.93	12.73	LAM		Clustered
1905	▪▪▪▪▪▪▪▪▪▪▪▪▪▪▪▪▪▪▪▪▪▪▪▪▪▪▪▪□□▪▪□□□□▪▪▪▪▪▪▪	777777777460771	2	1.41	9.52	T1		Clustered
2375	▪▪▪▪▪□▪▪▪▪▪▪▪▪▪▪▪▪▪▪▪▪▪▪▪□□□□□□▪□□□□▪▪▪▪▪▪▪	767777774020771	2	1.41	15.38	H1		Clustered
2502	▪▪▪▪▪▪▪▪▪▪▪▪▪▪▪▪▪▪▪□□□□□▪▪▪▪□▪▪▪□□□□▪▪▪▪▪▪▪	777777407560771	2	1.41	40	LAM6		Clustered
2940	▪▪▪▪▪▪▪▪▪▪▪▪▪▪▪▪▪▪▪▪□□□□▪▪▪▪▪▪▪▪□□□□▪□□▪▪▪▪	777777607760471	1	0.7	14.29	LAM9		Unique
3001	▪▪▪▪▪▪▪▪▪▪▪▪▪▪▪▪□□□□□▪▪▪▪▪▪□□□□▪□□□□▪▪▪▪▪▪▪	777774077020771	11	7.75	55	H3		Clustered
**3777**	▪▪▪▪▪▪▪▪▪▪▪▪▪▪▪▪□▪□□□▪▪▪▪▪▪▪□▪▪▪□□□□▪▪▪▪▪▪▪	777775077560771	1	0.7	50	T1	This study (n = 1), ESP (n = 1)	Unique
**3778**	▪▪▪▪▪▪▪▪▪▪▪▪▪▪▪▪□▪□□□▪▪▪▪▪▪□□□□▪□□□□▪▪▪▪▪▪▪	777775077020771	5	3.52	100	H3	This study (n = 5)	Clustered
**3779**	▪▪▪▪▪□▪▪▪▪▪▪▪▪▪▪□□□□□▪▪▪▪▪▪□□□□▪□□□□▪▪▪▪▪▪▪	767774077020771	2	1.41	100	H3	This study (n = 2)	Clustered
**3780**	▪▪▪□□□□□□□□□□▪▪▪▪□▪▪▪▪▪▪▪▪▪▪▪▪▪□□□□□▪▪▪▪▪▪▪	700036777740771	1	0.7	50	X3	This study (n = 1), PER (n = 1)	Unique

*A total of 22/26 SITs (n = 128) matched a preexisting shared-type in the database, whereas 4/26 SITs (n = 9 isolates) were newly created either within the present study or after a match with an orphan in the database.

A total of 17 SITs containing 128 isolates were clustered within this study (2 to 21 isolates per cluster), while 9 SITs contained a unique strain within this study. Note that SITs in bold indicates “newly created shared-type” (n = 4 containing 9 isolates) due to 2 or more strains belonging to an identical new pattern within this study or after a match with an orphan in the database.

**Lineage designations according to SITVIT2; “Unknown” designates patterns with signatures that do not belong to any of the major clades described in the database.

*** SIT designations are indicated in the table.

ESP = Spain, PER = Peru.

¥ Clustered strains correspond to a similar spoligotype pattern shared by 2 or more strains “within this study”; as opposed to unique strains harboring a spoligotype pattern that does not match with another strain from this study.

One hundred and twenty eight isolates, collected in Lima and different departments, were categorized into 26 shared-types (**Table 2**). Nine strains exhibited unique SIT patterns. The remaining isolates formed 17 different clusters. SIT50 (H3 clade) and SIT219 (T1 clade) were the predominant patterns - each pattern was presented in 21 different isolates, each one accounting for 14.79% of all isolates in the study (**Table 2**). Thirteen predominant SITs representing 120 strains were identified and their worldwide distribution was determined (**Table 3**). As mentioned previously, SIT50-H3 and SIT219-T1 were the predominant type (each with 14.79% of isolates in our study), followed by SIT47-H1 (11.27%), SIT1-Beijing (9.15%), SIT3001-H3 (7.75%), SIT53-T1 and SIT1355-LAM represented 4.93% each. Finally, SIT469-LAM1 and SIT3778-H3; SIT42-LAM9 and SIT49-H3 and SIT52-T2 and SIT93-LAM5 accounted for 3.52%, 2.82% and 2.11% respectively (**Table 2**). The discriminative power of the spoligotyping method, measured by the Hunter-Gaston index, was 0.924

**Table 3:** Description of clusters composed of predominant shared types (defined as SITs representing>2% strains, n = 13) in our study and their worldwide distribution in the SITVIT2 database.

SIT	Spoligotype Description	Octal Number	Number (%) in study	% in study vs. SITVIT2	Lineage	Distribution in Regions with> = 3% of a given SIT*	Distribution in countries with> = 3% of a given SIT**
1	□□□□□□□□□□□□□□□□□□□□□□□□□□□□□□□□□□▪▪▪▪▪▪▪▪▪	000000000003771	13 (9.15)	0.13	Beijing	ASIA-E 33.16, AMER-N 20.35, ASIA-SE 10.19, AFRI-S 8.37, ASIA-N 6.99, ASIA-S 5.01, EURO-N 3.13	USA 20.01, CHN 19.16, JPN 11.63, ZAF 8.37, RUS 6.99, VNM 3.93
42	▪▪▪▪▪▪▪▪▪▪▪▪▪▪▪▪▪▪▪▪□□□□▪▪▪▪▪▪▪▪□□□□▪▪▪▪▪▪▪	777777607760771	4 (2.82)	0.12	LAM9	AMER-S 30.36, AMER-N 14.2, EURO-S 10.41, EURO-W 9.85, AFRI-N 8.92, EURO-N 3.88, AFRI-E 3.7, AFRI-S 3.29	BRA 12.46, USA 12.34, COL 7.92, MAR 7.33, ITA 5.44, FXX 5.25, ESP 3.48, VEN 3.45, ZAF 3.29
47	▪▪▪▪▪▪▪▪▪▪▪▪▪▪▪▪▪▪▪▪▪▪▪▪▪□□□□□□▪□□□□▪▪▪▪▪▪▪	777777774020771	16 (11.27)	1.07	H1	EURO-W 21.34, AMER-N 17.99, EURO-S 14.16, AMER-S 12.95, EURO-E 7.58, EURO-N 6.64, ASIA-W 3.89, AFRI-N 3.83	USA 15.97, ITA 8.66, AUT 8.46, BRA 7.38, FXX 6.98, CZE 3.96, ESP 3.76, SWE 3.56, PER 3.22
49	▪▪▪▪▪▪▪▪▪▪▪▪▪▪▪▪▪▪▪▪▪▪▪▪▪▪▪▪▪▪□▪□□□□▪▪▪□▪▪▪	777777777720731	4 (2.82)	2.26	H3	EURO-N 23.73, EURO-W 18.08, AMER-N 16.95, AMER-S 12.99, EURO-S 11.86, AFRI-M 4.52	USA 14.69, FIN 12.99, FXX 11.86, SWE 10.73, PER 7.35, ITA 6.78, PRT 3.96, AUT 3.96
50	▪▪▪▪▪▪▪▪▪▪▪▪▪▪▪▪▪▪▪▪▪▪▪▪▪▪▪▪▪▪□▪□□□□▪▪▪▪▪▪▪	777777777720771	21 (14.79)	0.63	H3	AMER-N 18.71, AMER-S 18.08, EURO-W 17.93, EURO-S 11.78, EURO-E 5.63, EURO-N 4.37, AFRI-N 4.34, AFRI-S 4.13, CARI 3.56, ASIA-W 3.01	USA 17.9, BRA 7.2, FXX 7.02, AUT 6.21, ITA 5.54, ESP 5.54, PER 4.82, ZAF 4.13, CZE 3.74
52	▪▪▪▪▪▪▪▪▪▪▪▪▪▪▪▪▪▪▪▪▪▪▪▪▪▪▪▪▪▪▪▪□□□□▪▪▪□▪▪▪	777777777760731	3 (2.11)	0.33	T2	EURO-W 20.13, ASIA-E 15.68, AMER-N 14.24, EURO-N 12.46, EURO-S 5.45, ASIA-W 5.34, EURO-E 4.45, AFRI-E 4.45, AFRI-M 3.67	CHN 12.13, USA 11.9, SWE 10.01, FXX 9.23, BEL 5.01, ITA 3.56, JPN 3.34, NLD 3.0
53	▪▪▪▪▪▪▪▪▪▪▪▪▪▪▪▪▪▪▪▪▪▪▪▪▪▪▪▪▪▪▪▪□□□□▪▪▪▪▪▪▪	777777777760771	7 (4.93)	0.12	T1	AMER-N 16.92, EURO-W 16.21, AMER-S 12.47, EURO-S 9.75, ASIA-W 7.13, EURO-N 5.53, AFRI-S 5.15, AFRI-E 4.67, ASIA-E 4.39, AFRI-N 3.65, EURO-E 3.38	USA 13.67, FXX 8.16, ITA 5.53, BRA 5.31, ZAF 5.03, TUR 3.6, AUT 3.55, CHN 3.2
93	▪▪▪▪▪▪▪▪▪▪▪▪□▪▪▪▪▪▪▪□□□□▪▪▪▪▪▪▪▪□□□□▪▪▪▪▪▪▪	777737607760771	3 (2.11)	0.84	LAM5	AMER-S 48.6, AMER-N 21.35, CARI 11.8, EURO-S 8.43, EURO-W 5.06	VEN 26.4, USA 21.07, BRA 12.08, ITA 5.34, GLP 5.34, PER 4.78, HTI 4.21, FXX 3.09, ESP 3.09
219	▪▪▪▪▪▪▪▪▪▪▪▪▪□□□□□▪▪▪▪▪▪▪▪▪▪▪▪▪▪□□□□▪▪▪▪▪▪▪	777740777760771	21 (14.79)	35.59	T1	AMER-N 52.54, AMER-S 47.46	USA 52.54, PER 47.46
469	□□□▪▪▪▪▪▪▪▪▪▪▪▪▪▪▪▪▪□□□□▪▪▪▪▪▪▪▪□□□□▪▪▪▪▪▪▪	077777607760771	5 (3.52)	14.71	LAM1	AMER-N 38.24, AMER-S 32.35, EURO-S 5.88, ASIA-SE 5.88, AFRI-N 5.88	USA 38.24, PER 26.47, ITA 5.88, EGY 5.88
1355	▪▪▪▪▪▪▪▪▪▪▪▪▪▪▪▪▪▪▪□□□□□▪▪▪▪□▪▪▪□□□□▪▪▪□▪▪▪	777777407560731	7 (4.93)	12.73	LAM	AMER-S 61.82, EURO-S 23.64, AMER-N 12.73	PER 60.0, ITA 18.18, USA 12.73, ESP 5.46
3001	▪▪▪▪▪▪▪▪▪▪▪▪▪▪▪▪□□□□□▪▪▪▪▪▪□□□□▪□□□□▪▪▪▪▪▪▪	777774077020771	11 (7.75)	55	H3	AMER-S 95.0, AMER-N 5.0	PER 95.0, USA 5.0
3778	▪▪▪▪▪▪▪▪▪▪▪▪▪▪▪▪□▪□□□▪▪▪▪▪▪□□□□▪□□□□▪▪▪▪▪▪▪	777775077020771	5 (3.52)	100	H3	AMER-S 100.0	PER 100.0

* Worldwide distribution is reported for regions with more than 3% of a given SITs as compared to their total number in the SITVIT2 database.

The definition of macro-geographical regions and sub-regions (http://unstats.un.org/unsd/methods/m49/m49regin.htm) is according to the United Nations; Regions: AFRI (Africa), AMER (Americas), ASIA (Asia), EURO (Europe), and OCE (Oceania), subdivided in: E (Eastern), M (Middle), C (Central), N (Northern), S (Southern), SE (South-Eastern), and W (Western). Furthermore, CARIB (Caribbean) belongs to Americas, while Oceania is subdivided in 4 sub-regions, AUST (Australasia), MEL (Melanesia), MIC (Micronesia), and POLY (Polynesia).

Note that in our classification scheme, Russia has been attributed a new sub-region by itself (Northern Asia) instead of including it among rest of the Eastern Europe. It reflects its geographical localization as well as due to the similarity of specific TB genotypes circulating in Russia (a majority of Beijing genotypes) with those prevalent in Central, Eastern and South-Eastern Asia.

** The 3 letter country codes are according to http://en.wikipedia.org/wiki/ISO_3166-1_alpha-3; countrywide distribution is only shown for SITs with ≥3% of a given SITs as compared to their total number in the SITVIT2 database.

### Analysis by MIRU-VNTRs

The results of the MIRU analysis (15-MIT) showed that the 142 isolates were classified into clustered (n = 49 or 34.5% grouped in 11 clusters) and unclustered (n = 93 or 65.5%) patterns. The clustered strains corresponded to following lineages by spoligotyping: Beijing (n = 4, 8.2%); Haarlem (n = 22, 44.9%); T (n = 14, 28.6%); LAM (n = 8, 16.3%) and an unknown lineage (n = 1, 2%) (**S2 Table**). The 93 unclustered isolates showed unique MIRU patterns, all except one (which corresponded to SIT49/MIT369 in the SITVIT2 database), the remaining patterns were not yet reported and corresponded to orphan patterns. A dendrogram was constructed based on both spoligotyping and MIRU results (**S1 Figure**), and showed that the isolates could be divided into three groups based on their phylogenetic clustering and genotypic characteristics. Groups I, II, and III contained 13, 47 and 82 isolates respectively. Group I presented one cluster (3 isolates), group II presented 4 clusters (12 isolates) and group III presented 6 clusters (19 isolates). We observed that fourteen isolates were not grouped in the dendrogram because all of them had a different spoligotype (**S1 Figure**) in regards to the cluster generated by MIRU-15 and the spoligotyping profile. The HGDI for MIRU-15 was 0.993

### Relationship between lineage of MTB and Peruvian XDR strains

**Figure 1:**
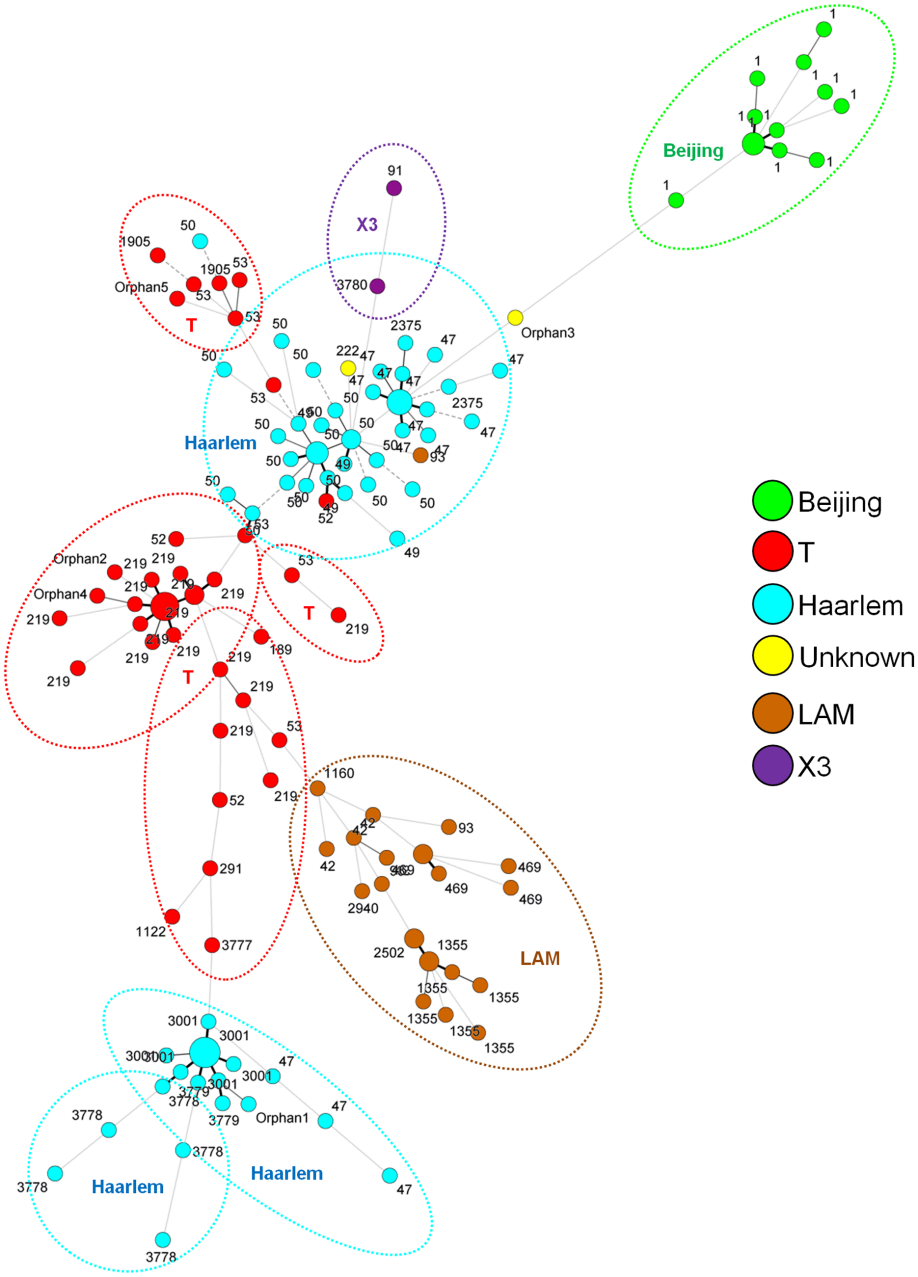
MST of spoligotyping in conjunction with MIRU-15 typing. Distinction of the genotypic lineages is shown by circles of different colors. Patterns colored in yellow indicate a strain with an unknown signature (unclassified in the SITVIT2 database). The MST allows a finer discrimination of the main spoligotype central nodes.

A minimum spanning tree based spoligotyping data was constructed to visualize the patterns connected with cities of isolation of XDR strains in Peru (**S2 Figure**). All the strains isolated in the departments of Peru were present in Lima except the SIT1122, orphan 2 and orphan 5 that were present exclusively in Callao, SIT1160 in Ica and SIT3779 in Callao and Junin. A composite MST based on both spoligotyping and MIRU results was drawn for a better discrimination of circulating lineages among XDR strains (**Fig. 1**), and allowed separation of strains in six well-defined groups around main spoligotype central nodes, comprised of Beijing, T, Haarlem, LAM, X3 and strains with unknown signatures. Among these identified lineages, all were well distributed; nonetheless one may notice the predominance of Haarlem group followed by T sublineages (**Fig. 1**).

We also drew a spoligoforest tree as a hierarchical layout, where the continuity of the lines indicates the weight of the hypothetical evolutionary relationship between spoligotypes (**S3 Figure**). In this illustration, each pattern from the study is represented by a node with area size being proportional to the total number of isolates. Changes (loss of spacers) are represented by directed edges between nodes, with the arrowheads pointing to descendant spoligotypes. Using this model, solid black lines link patterns that are very similar, i.e., loss of one spacer only as opposed to dashed and dotted lines that represent respectively 2 or more spacer changes. The spoligoforest obtained showed four subtrees with connected components and two unconnected nodes. One may notice that SIT50/H3 and SIT219/T1 are the biggest nodes (n = 21 each), followed by SIT47/H1 (n = 16), SIT1/Beijing (n = 13), SIT3001/H3 (n = 11), SIT53/T1 and SIT1355/LAM (n = 7 each); followed by smaller nodes of 5 strains and less. Eighteen spoligotypes descended from SIT53, six of which are in small clusters (range 1–4), and two in larger clusters (21 isolates) - one from a lineage distinct to SIT53. The hypothetical evolutionary relationship between spoligotypes SIT53 and SIT50 and SIT53 and SIT219, the spoligotypes with the largest clusters in the data, were strong and weak respectively. Five other spoligotypes (SIT52, SIT189, SIT291, SIT1905 and SIT1122) also showed a strong relationship with SIT53, while two other subtrees (rooted by spoligotype SIT3777 and SIT2502) lead to Haarlem and LAM lineage strains. Globally, this analysis suggested that SIT53/T1 was at the origin of many of the T lineage strains as well as a big proportion of Haarlem lineage strains (SIT50/H3, followed by SIT47/H1, SIT49/H3, and SIT2375/H1), as opposed to the SIT1/Beijing strains that did not appear to evolve into minor Beijing sublineages among the XDR-TB strains.

### Association between of Peruvian XDR strains and resistance to injectable drugs

We drew a spoligotyping based MST to visualize a possible link between lineages and treatment history of the patients (**S4 Figure**). It shows that a majority of XDR-TB cases concerned relapsed patients (n = 129, 90.85%) with only rare new cases (n = 13, 9.15%); the latter concerned SIT3001/H3, SIT3778/H3, SIT50/H3, SIT 47/H1, SIT219/T1, SIT 1160/LAM5, and SIT1/Beijing. Regarding drug-resistance to injectable drugs CAP and KAN (explained earlier, see also **S2 Table**), the following distribution patterns were noticed: CAP alone, n = 22/142 (15.5%); KAN alone, n = 31/142 (21.8%); and both CAP+KAN, n = 89/142 (62.7%). Interestingly, the MST showed that only 5 shared-types (SIT50, 53, 219, 3001 and 3778) contained all the 3 patterns of drug resistance observed. Among the remaining cases, 4 shared-types (SIT1, 52, 93, and 49) contained strains with 2 drug resistance patterns (KAN-R and CAP+KAN both), while 2 shared-types (SIT47, 1355) strains with CAP-R and CAP+KAN.

### Evolutionary relationships between Beijing lineages isolated from Peruvian XDR strains

**Figure 2:**
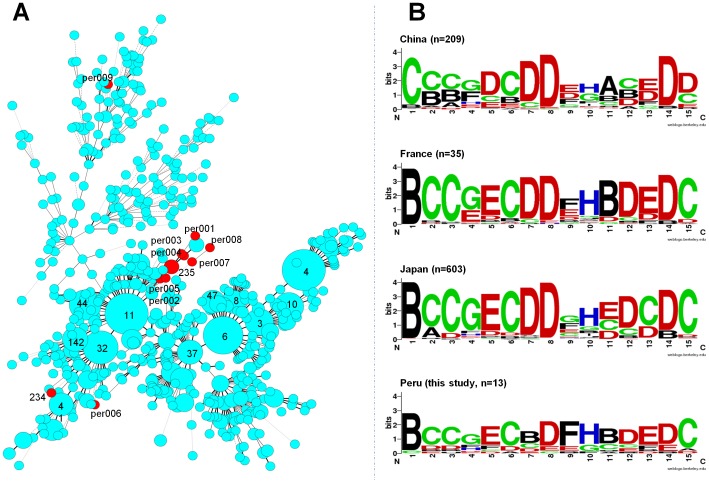
Evolutionary relationships between Beijing lineages isolated from Peruvian XDR-TB strains (n = 13) vs. other countries in the SITVIT2 database (n = 863). (A). A minimum spanning tree based on 15-loci MIRU-VNTR of the Peruvian Beijing isolates (highlighted in red) versus isolates from other countries (shown in cyan-blue color). (B). Comparative diversity of Peruvian Beijing vs. other countries using the WebLogo application. Each logo consists of stacks of symbols, one stack for each MIRU loci. The overall height of the stack indicates the conservation of a given MIRU loci with a fixed number of copies at that position (i.e., if 100% of the strains conserve the same number of copies for a given MIRU loci, it corresponds to 4 bits), while the height of individual symbols within the stack indicates the relative frequency of number of copies of a given MIRU loci at that position. WebLogo stack label/number of copies for MIRU loci: A/1, B/2, C/3, D/4, E/5, F/6, G/7, H/8, I/9, J/10, K/11, L/12, M/13, N/14, O/15, P/16, Q/0, U/Unknown.

A MIRU based MST (**Fig. 2A**) and WebLogo (**Fig. 2B**) were drawn to compare the Beijing lineage *M. tuberculosis* strains encountered in our study (n = 13) vs. other countries in the SITVIT2 database (n = 863) for which a significant number of strains with 15-loci typing data (35 or more) were available: Japan n = 603, China n = 209 and France n = 35. The results obtained showed that a majority of the Peruvian strains have a specific phylogenetic position on the MST, close to one of the predominant MIRU-15 International Type-11 (15-MIT11), found in Japan (**Fig. 2A**). However, 3 Beijing strains from Peru are well isolated from others: 2 orphans per006 and per009 and a shared-type strain MIT234. As illustrated in **Fig. 2A**, per006 and 15-MIT234 are phylogenetically close to Japanese Beijing strains, while per009 at the top of the MST is close to Chinese patterns. The WebLogo representation (**Fig. 2B**) of each stack of symbols corresponding to each MIRU loci indicates that each region (Japan, China, France or Peru) has some specificities, but despite this fact, some correlation and similarity can be seen, notably between the Japanese, French and Peruvian (this study) strains. Despite these similarities, we can distinguish the Peruvian MIRU-15 isolates by its particular specific variation of number of copies on the 7^th^, 9^th^ and 11^th^ loci positions. However, the statistical analysis based on the WebLogo data (data not shown) did not yield statistically significant variations by Fisher's Exact Test, essentially because of the small sample size of the Peruvian Beijing strains.

## Discussion

TB is a prevalent disease in Peru. Since 2009 the government declared the disease as sanitary emergency due to the constant increase in the number of TB cases, mainly MDR-TB cases and the emergence of XDR-TB [19]. The distribution of TB cases is not homogeneous in Peru; the central coast (mainly in Lima) is the setting which presents the 90% of all TB cases and 96.7% of MDR-TB cases [19]. Since the first cases of XDR-TB were detected in Peru in 2007 [3], their number has been constantly increasing. Subsequent emergence of primary XDR-TB cases in children [4] has further worsened the situation, and controlling XDR-TB presents today a formidable challenge for the public health system. However, there is little information concerning the molecular epidemiology and genotypic diversity of circulating XDR-TB clones in Peru. There were only very few Peruvian strains reported in the previous versions of the databases including in SITVITWEB [11]. However, thanks to recent studies [20], [21],[22], and the fact that the number of Peruvian strains was considerably increased in the updated SITVIT2 database (to almost 900), we thought it desirable to investigate the genetic diversity of XDR-TB isolates from Peru.

In the present study we analyzed 142 XDR-TB isolates which were grouped into 17 clusters. With the exception of 2 strains (1.41%) that did not match lineages reported so far, the remaining strains were distributed among following lineages: Haarlem (43.66%), T (27.46%), LAM (16.2%), Beijing (9.15%), and X clade (1.41%). Spoligotype led to a clustering for 90.1% strains vs. 34.5% by 15-loci MIRUs. Furthermore, 9 isolates (6.3%) were recorded as new SITs and 5 isolates (3.5%) corresponded to orphan patterns.

A recent study reported that the predominant genotypes in susceptible and resistant MTB isolates from Peru were LAM (23.8%), T (23.8%), Haarlem (22.3%), and Beijing (9.3%). Forty-three isolates were not reported previously (13.3%). The author concluded that the relatively high number of clusters suggests that recent transmission may be one major cause of the high incidence of TB in Peru [22]. Other studies carried out in Venezuela [23], [24], Paraguay [25], Honduras [26], and Brazil [27] showed similar results with LAM being the most prevalent lineage. For example, the predominant MTBC lineages in Brazil in decreasing order were: LAM (46%); the ill-defined T (18.6%); the Haarlem (12.2%), the X (4.7%), the S (1.9%), and the East African Indian (EAI) (0.85%) families [27]. Interestingly, this descriptive information of MTBC lineages/sublineages differs from our results on XDR isolates in Peru, where Haarlem was the most prevalent lineage, followed by T, LAM, and Beijing. One may notice that both SIT219/T1 and SIT50/H3 are the two most predominant SITs in our study: 14.8% each (**Table 2**), are also predominant in USA (**Table 3**). However, as opposed to SIT50/H3 which is equally present in North, Central and South America (between 4 to 6%), the SIT219/T1 is almost exclusively found in Peru (p<0.001), and to a lesser extent in North America (**S3 Table**). This observation may indirectly suggest that the strain was brought to the US by Peruvian migrants.

A study by Dalla Costa et.al [28] showed that Haarlem sublineage, mainly SIT50/H3, had a high frequency of *katG* S315T mutation in INH resistant MTB strains. Furthermore, it has been reported [29] that this sublineage presents mutations in certain genes allowing greater adaptability to hostile environments, such as those present following challenge by anti-TB drugs or engulfment by macrophages. These characteristics may partially explain the successful spread of Haarlem lineage strains, often associated with drug-resistance outbreaks in South America and elsewhere [28]. Though this explanation alone might not be sufficient to clarify the high prevalence of Haarlem sublineage in our study, mainly SIT50/H3 lineage strains, it could be partially responsible for the high prevalence of MDR-TB and XDR-TB cases observed. Future investigations should ideally focus on these aspects in Peru by comparing the relationship between Haarlem and other sublineages versus drug resistance mutations.

There is scarce information about genotyping studies in XDR isolates; in 2008 the first description of XDR genotypes concerned cases observed in South Africa [30]. Out of 41 isolates genotyped by spoligotyping, thirty-one isolates matched a previously described spoligotype; among these, the Beijing lineage was the largest group (34%) followed by LAM, EAI, T, Haarlem, S and X3 sublineages. Another study done in Colombia characterized 10 XDR isolates [31] which were identified as SIT190/Beijing, SIT62/H1, SIT881/unknown, SIT545/LAM2 and SIT3010/S. Surprisingly, our results showed that the Beijing lineage is not predominant among Peruvian XDR isolates since the proportion of SIT1/Beijing strains in our study (9.15%) actually matches with the proportion of Beijing strains in other recently published studies from Peru [32], [33]. Ritacco et al. [33] speculated that the Beijing family strains were first introduced into Peru, and eventually into other South American countries, when Peru received a significant number of Chinese immigrants in the mid-19th century. In this context, our results using MST and WebLogo analysis shows that Peruvian Beijing XDR strains are more related to Japanese strains than Chinese strains (**Fig. 2**). This finding is possible because Peru also received Japanese immigrants at the end of the 19^th^ century. A previous study [32] together with our results suggests the co-circulation of Beijing family with Japanese and Chinese ancestors in Peru.

In regards to the SIT distribution and the gender of patients, our study found that the difference between males and females was significant (p-value = 0.039). We noticed that the proportion of SIT47/H1 was particularly important among females. The proportions of SIT1355/LAM, SIT3001/H3, and SIT3778/H3 were notably more important among male patients. Although the exact reasons are not well understood, these results might underline a preference for certain sublineages between male and female patients; nonetheless these differences should be verified in future analyses.

We also found that the XDR-TB affected slightly more females than males in our study (**Table 1**). This appears unusual since males are usually more frequently affected by TB than females, probably because of a higher exposition to various well-known risk factors. However, as high as 90% of the XDR-TB cases in our study concerned relapse cases, there is a possibility that the higher frequency of XDR-TB in female patients was due to a greater likelihood of treatment abandonment.

In Peru, the HIV cases are restricted to risk groups at difference to other countries. Pulmonary tuberculosis cases (susceptible, MDR and XDR tuberculosis) appear in general population. This is the reason for which HIV associated to tuberculosis appears in very low proportion (<3% of total TB cases in Peru, source: Ministry of Health). In our study, none of the 142 patients were HIV-positive. We may also mention that of our 227 cryopreserved samples, 2 samples were isolated from HIV-positive patients that unfortunately could not be reactivated upon subculturing.

In regards to the treatment history of patients, the majority were relapsed cases with a long history of treatment, initially for susceptible and then MDR-TB. The new cases almost always concerned predominant SITs (with the only exception of SIT1160), suggesting that MTBC isolates with acquired drug-resistance from retreated patients might be actively being transmitted to newly infected patients (primary XDR-TB cases).

We must nonetheless acknowledge limitations of the data presented here. Most importantly, our sampling strategy was opportunistic making use of a strain bank, and the period of sampling was relatively short (2.5 years). However, since XDR-TB cases represent 6% of MDR-TB cases, we considered that these cumulative cases of XDR strains over a period of 2.5 years might be considered as being representative of Peruvian XDR strains. There are advantages in having a strain bank which can be used for genotyping studies when epidemiological data are delinked from patient identifiers, but it also leads to obvious drawbacks, e.g., only limited clinical data are available and returning to clinical notes for further detail is not possible.

In conclusion, our study report for the first time in Peru, the genetic characterization and evolutionary relationships of XDR-TB strains, and highlights a significant proportion of Haarlem sublineage, followed by T sublineages – which are not among the usually predominant lineages in Peru. Furthermore, against all odds – we did not find Beijing lineage strains as the major cause of prevailing XDR-TB cases in Peru. Further studies are necessary to corroborate these results and to investigate whether these lineages continue to be a major cause of XDR-TB in Peru.

## Supporting Information

S1 Figure Dendrogram of Peruvian XDR-TB strains generated by MIRU-VNTRplus software (www.miru-vntrplus.org). The dendrogram shows three groups (I, II, III) containing 11clusters (n = 34 strains; see text for details).
(PDF)

S2 Figure A minimum spanning tree illustrating the relationships between spoligotype patterns and the cities of isolation of the strains.
(TIF)

S3 Figure A spoligoforest tree drawn as Hierarchical Layout showing the parent to descendant relationships of the *M. tuberculosis* spoligotypes of Peruvian XDR isolates. The heuristic used selects a single inbound edge with a maximum weight using a Zipf model; solid black lines link patterns that are very similar, i.e., loss of one spacer only (maximum weigh being 1.0), while dashed lines represent links of weight comprised between 0.5 and 1, and dotted lines a weight less than 0.5. Note that orphan isolates (double circled), either appear at terminal positions on the tree, or as isolated strain without interconnections with the other.
(TIF)

S4 Figure A minimum spanning tree illustrating the relationships between spoligotype patterns and the treatment history of patients.
(TIF)

S1 Table Descriptive statistics on age of patients.
(PDF)

S2 Table Detailed genotyping and drug-resistance data and demographic information on *M. tuberculosis* XDR strains (n = 142) isolated from adults with pulmonary tuberculosis in Peru.
(PDF)

S3 Table A comparison of the proportion of all SITs found in this study as compared to the other strains isolated in Peru and neighboring regions (Northern America, Southern America, Central America and Caribbean), recorded in the SITVIT2 database.
(PDF)
