# Supplementary material for: Characterization of the Genetic Diversity of Extensively-Drug Resistant Mycobacterium tuberculosis Clinical Isolates from Pulmonary Tuberculosis Patients in Peru
Source: PLoS One. 2014 Dec 9;9(12):e112789. doi: 10.1371/journal.pone.0112789 (PMC4260790; doi:10.1371/journal.pone.0112789)

Figure S1. Dendrogram of Peruvian M. tuberculosis XDR strains generated by MIRU-VNTRplus software (www.miru-vntrplus.org). The dendrogram shows three groups (I, II, III) which grouped 11 clusters containing 34 strains (See text)

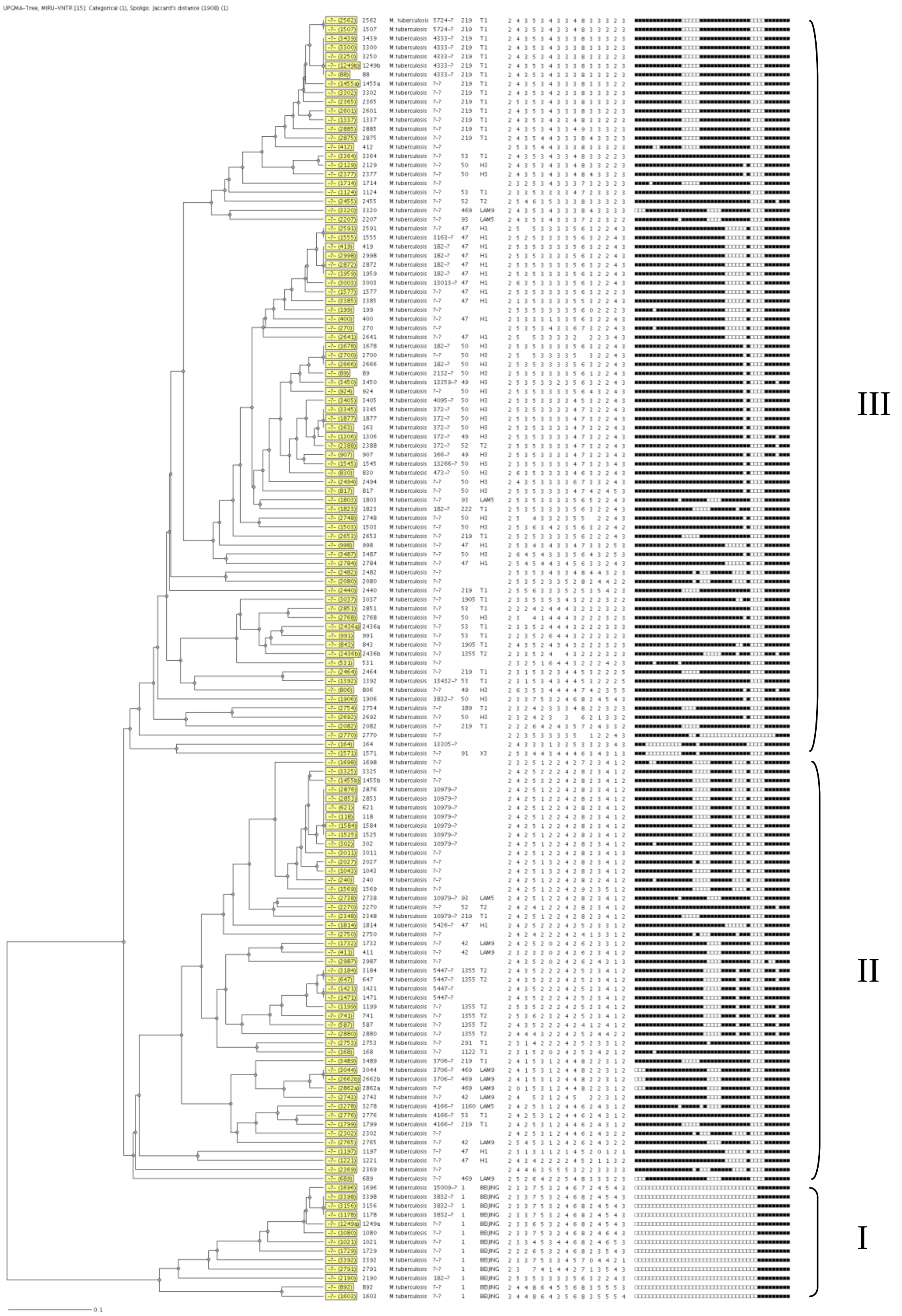

Supplement: S1 Figure — Dendrogram of Peruvian XDR-TB strains generated by MIRU-VNTRplus software (www.miru-vntrplus.org). The dendrogram shows three groups (I, II, III) containing 11clusters (n = 34 strains; see text for details). (PDF) [file pone.0112789.s001.pdf]
