# Supplementary figures and images for: Characterization of the Genetic Diversity of Extensively-Drug Resistant Mycobacterium tuberculosis Clinical Isolates from Pulmonary Tuberculosis Patients in Peru
Source: PLoS One. 2014 Dec 9;9(12):e112789. doi: 10.1371/journal.pone.0112789 (PMC4260790; doi:10.1371/journal.pone.0112789)

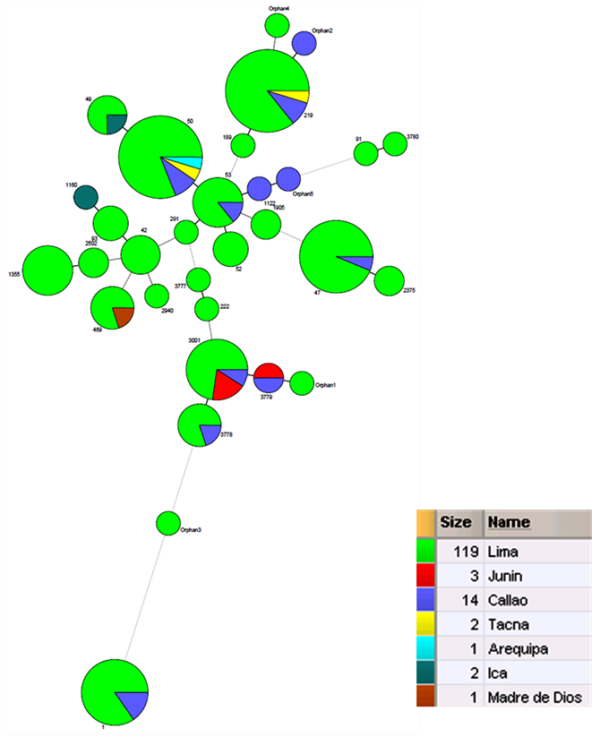

Supplement: S2 Figure — A minimum spanning tree illustrating the relationships between spoligotype patterns and the cities of isolation of the strains. (TIF) [file pone.0112789.s002.tif]

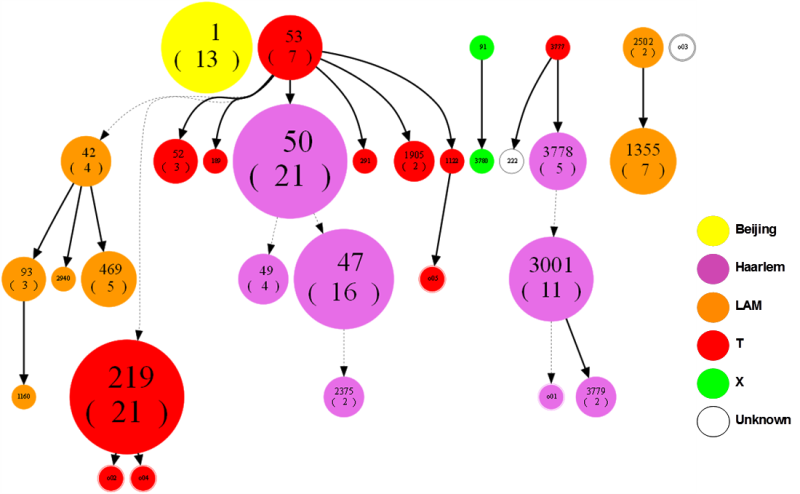

Supplement: S3 Figure — A spoligoforest tree drawn as Hierarchical Layout showing the parent to descendant relationships of the M. tuberculosis spoligotypes of Peruvian XDR isolates. The heuristic used selects a single inbound edge with a maximum weight using a Zipf model; solid black lines link patterns that are very similar, i.e., loss of one spacer only (maximum weigh being 1.0), while dashed lines represent links of weight comprised between 0.5 and 1, and dotted lines a weight less than 0.5. Note that orphan isolates (double circled), either appear at terminal positions on the tree, or as isolated strain without interconnections with the other. (TIF) [file pone.0112789.s003.tif]

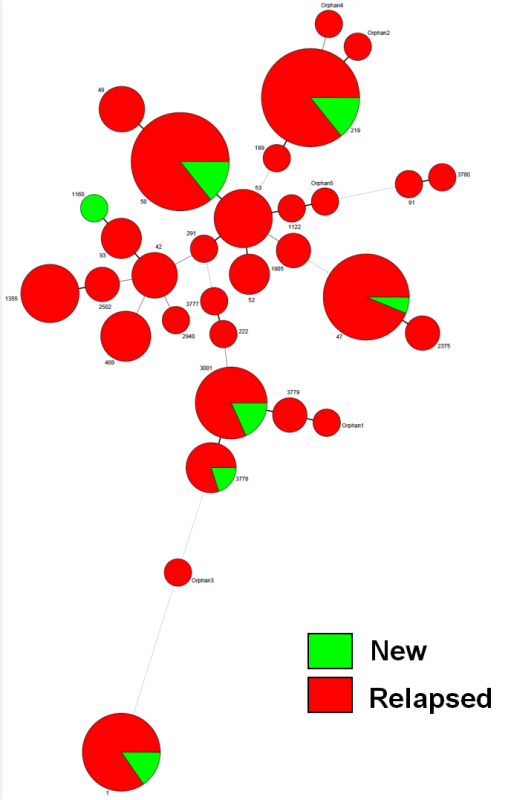

Supplement: S4 Figure — A minimum spanning tree illustrating the relationships between spoligotype patterns and the treatment history of patients. (TIF) [file pone.0112789.s004.tif]
