# Supplementary material for: Characterization of the Genetic Diversity of Extensively-Drug Resistant Mycobacterium tuberculosis Clinical Isolates from Pulmonary Tuberculosis Patients in Peru
Source: PLoS One. 2014 Dec 9;9(12):e112789. doi: 10.1371/journal.pone.0112789 (PMC4260790; doi:10.1371/journal.pone.0112789)
Supplement: S1 Table — Descriptive statistics on age of patients. (PDF) [file pone.0112789.s005.pdf]

<u>Supplemental Table S1</u>: Descriptive statistics on age of patients

	Male patients	*Female patients*	***Total***
Count	90	52	**142**
Mean	36.33	30.06	**34.04**
Sample variance	180.49	108.88	**162.52**
Sample standard deviation	13.43	10.43	**12.75**
Minimum	15	17	**15**
Maximum	72	70	**72**
Range	57	53	**57**
1st quartile	26.00	23.00	**24.00**
Median	34.00	26.00	**31.00**
3rd quartile	46.50	34.00	**42.00**
Interquartile range	20.50	11.00	**18.00**
Mode	40.00	24.00	**24.00**
