# Supplementary material for: Characterization of the Genetic Diversity of Extensively-Drug Resistant Mycobacterium tuberculosis Clinical Isolates from Pulmonary Tuberculosis Patients in Peru
Source: PLoS One. 2014 Dec 9;9(12):e112789. doi: 10.1371/journal.pone.0112789 (PMC4260790; doi:10.1371/journal.pone.0112789)
Supplement: S2 Table — Detailed genotyping and drug-resistance data and demographic information on M. tuberculosis XDR strains (n = 142) isolated from adults with pulmonary tuberculosis in Peru. (PDF) [file pone.0112789.s006.pdf]

Supplemental Table S2: Detailed genotyping and drug-resistance data and demographic information on cryopreserved M. tuberculosis XDR strains (n=142) isolated from adults with pulmonary tuberculosis in Peru.

IsoNumber	Year	Strain	Sex/age	Isolation city	Spoligotype Description	Octal code	Clade	SIT	MIRU15	15-MIT	Drug-R to CAP or KAN or both	Patient status
PER0620094892	2009	892	F/41	Callao	□□□□□□□□□□□□□□□□□□□□□□□□□□□□□□□□□□■■■■■■■■■	000000000003771	Beijing	1	244864556835553	Orph007	CAP S+KAN R	Relapsed
PER06200941021	2009	1021	M/20	Lima	□□□□□□□□□□□□□□□□□□□□□□□□□□□□□□□□□□■■■■■■■■■	000000000003771	Beijing	1	233453446824653	Orph002	CAP S+KAN R	Relapsed
PER06200941080	2009	1080	M/26	Lima	□□□□□□□□□□□□□□□□□□□□□□□□□□□□□□□□□□■■■■■■■■■	000000000003771	Beijing	1	233753246824643	Orph005	CAP S+KAN R	Relapsed
PER06200941178	2009	1178	F/25	Lima	□□□□□□□□□□□□□□□□□□□□□□□□□□□□□□□□□□■■■■■■■■■	000000000003771	Beijing	1	233753246824543	235	CAP S+KAN R	Relapsed
PER06200941603	2009	1603	F/20	Lima	□□□□□□□□□□□□□□□□□□□□□□□□□□□□□□□□□□■■■■■■■■■	000000000003771	Beijing	1	344864356835554	Orph008	CAP S+KAN R	Relapsed
PER06200741696	2007	1696	M/25	Lima	□□□□□□□□□□□□□□□□□□□□□□□□□□□□□□□□□□■■■■■■■■■	000000000003771	Beijing	1	233753246724543	Orph004	CAP R+KAN R	Relapsed
PER06200741729	2007	1729	M/40	Lima	□□□□□□□□□□□□□□□□□□□□□□□□□□□□□□□□□□■■■■■■■■■	000000000003771	Beijing	1	222653246823543	Orph001	CAP S+KAN R	Relapsed
PER06200842190	2008	2190	M/50	Lima	□□□□□□□□□□□□□□□□□□□□□□□□□□□□□□□□□□■■■■■■■■■	000000000003771	Beijing	1	253533335632243	234	CAP R+KAN R	New
PER06200842791	2008	2791	M/19	Callao	□□□□□□□□□□□□□□□□□□□□□□□□□□□□□□□□□□■■■■■■■■■	000000000003771	Beijing	1	23-741442713543	Orph009	CAP S+KAN R	Relapsed
PER06200843156	2008	3156	M/40	Lima	□□□□□□□□□□□□□□□□□□□□□□□□□□□□□□□□□□■■■■■■■■■	000000000003771	Beijing	1	233753246824543	235	CAP S+KAN R	Relapsed
PER06200843392	2008	3392	F/24	Lima	□□□□□□□□□□□□□□□□□□□□□□□□□□□□□□□□□□■■■■■■■■■	000000000003771	Beijing	1	233753345704421	Orph006	CAP S+KAN R	Relapsed
PER06200843398	2008	3398	M/28	Lima	□□□□□□□□□□□□□□□□□□□□□□□□□□□□□□□□□□■■■■■■■■■	000000000003771	Beijing	1	233753246824543	235	CAP R+KAN R	Relapsed
PER06200741249a	2007	1249a	M/50	Lima	□□□□□□□□□□□□□□□□□□□□□□□□□□□□□□□□□□■■■■■■■■■	000000000003771	Beijing	1	233653246824543	Orph003	CAP R+KAN R	New
PER0620094411	2009	411	M/40	Lima	■■■■■■■■■■■■■■■■■■■■□□□□■■■■■■■■□□□□■■■■■■■	777777607760771	LAM9	42	232320242623412	Orph010	CAP R+KAN R	Relapsed
PER06200741732	2007	1732	F/44	Lima	■■■■■■■■■■■■■■■■■■■■□□□□■■■■■■■■□□□□■■■■■■■	777777607760771	LAM9	42	242520242623312	Orph011	CAP R+KAN R	Relapsed
PER06200842743	2008	2743	F/34	Lima	■■■■■■■■■■■■■■■■■■■■□□□□■■■■■■■■□□□□■■■■■■■	777777607760771	LAM9	42	24-531245-22312	Orph013	CAP R+KAN R	Relapsed
PER06200842765	2008	2765	F/56	Lima	■■■■■■■■■■■■■■■■■■■■□□□□■■■■■■■■□□□□■■■■■■■	777777607760771	LAM9	42	254531242624322	Orph012	CAP R+KAN R	Relapsed
PER0620074400	2007	400	F/22	Lima	■■■■■■■■■■■■■■■■■■■■■■■■■□□□□□□■□□□□■■■■■■■	777777774020771	H1	47	233331335632243	Orph016	CAP R+KAN S	Relapsed
PER0620074419	2007	419	F/26	Lima	■■■■■■■■■■■■■■■■■■■■■■■■■□□□□□□■□□□□■■■■■■■	777777774020771	H1	47	253533335632243	234	CAP R+KAN R	Relapsed
PER0620094998	2009	998	F/40	Callao	■■■■■■■■■■■■■■■■■■■■■■■■■□□□□□□■□□□□■■■■■■■	777777774020771	H1	47	253434334733253	Orph020	CAP R+KAN R	Relapsed
PER06200741197	2007	1197	F/24	Lima	■■■■■■■■■■■■■■■■■■■■■■■■■□□□□□□■□□□□■■■■■■■	777777774020771	H1	47	231311214520121	Orph015	CAP R+KAN S	Relapsed
PER06200741221	2007	1221	F/22	Lima	■■■■■■■■■■■■■■■■■■■■■■■■■□□□□□□■□□□□■■■■■■■	777777774020771	H1	47	243422224521132	Orph018	CAP R+KAN S	Relapsed
PER06200741555	2007	1555	F/26	Lima	■■■■■■■■■■■■■■■■■■■■■■■■■□□□□□□■□□□□■■■■■■■	777777774020771	H1	47	252533335632243	Orph019	CAP R+KAN R	Relapsed
PER06200941577	2009	1577	M/53	Lima	■■■■■■■■■■■■■■■■■■■■■■■■■□□□□□□■□□□□■■■■■■■	777777774020771	H1	47	253533335632223	Orph021	CAP R+KAN R	Relapsed
PER06200741814	2007	1814	M/41	Lima	■■■■■■■■■■■■■■■■■■■■■■■■■□□□□□□■□□□□■■■■■■■	777777774020771	H1	47	242522242523312	Orph017	CAP R+KAN R	Relapsed
PER06200841959	2008	1959	F/25	Lima	■■■■■■■■■■■■■■■■■■■■■■■■■□□□□□□■□□□□■■■■■■■	777777774020771	H1	47	253533335632243	234	CAP R+KAN S	Relapsed
PER06200842591	2008	2591	F/24	Lima	■■■■■■■■■■■■■■■■■■■■■■■■■□□□□□□■□□□□■■■■■■■	777777774020771	H1	47	25-533335632243	Orph025	CAP R+KAN S	Relapsed
PER06200842641	2008	2641	M/43	Lima	■■■■■■■■■■■■■■■■■■■■■■■■■□□□□□□■□□□□■■■■■■■	777777774020771	H1	47	25-533332-22343	Orph024	CAP R+KAN S	Relapsed
PER06200842784	2008	2784	F/36	Lima	■■■■■■■■■■■■■■■■■■■■■■■■■□□□□□□■□□□□■■■■■■■	777777774020771	H1	47	254544345633243	Orph022	CAP R+KAN S	New
PER06200842872	2008	2872	F/26	Lima	■■■■■■■■■■■■■■■■■■■■■■■■■□□□□□□■□□□□■■■■■■■	777777774020771	H1	47	253533335632243	234	CAP R+KAN R	Relapsed
PER06200842998	2008	2998	M/24	Lima	■■■■■■■■■■■■■■■■■■■■■■■■■□□□□□□■□□□□■■■■■■■	777777774020771	H1	47	253533335632243	234	CAP R+KAN S	Relapsed
PER06200843003	2008	3003	F/22	Lima	■■■■■■■■■■■■■■■■■■■■■■■■■□□□□□□■□□□□■■■■■■■	777777774020771	H1	47	263533335632243	Orph023	CAP R+KAN S	Relapsed
PER06200843385	2008	3385	F/25	Lima	■■■■■■■■■■■■■■■■■■■■■■■■■□□□□□□■□□□□■■■■■■■	777777774020771	H1	47	213533335532243	Orph014	CAP R+KAN S	Relapsed
PER0620094806	2009	806	M/47	Lima	■■■■■■■■■■■■■■■■■■■■■■■■■■■■■■□■□□□□■■■□■■■	777777777720731	H3	49	263534444742355	Orph027	CAP S+KAN R	Relapsed
PER0620094907	2009	907	M/15	Ica	■■■■■■■■■■■■■■■■■■■■■■■■■■■■■■□■□□□□■■■□■■■	777777777720731	H3	49	253533334732343	369	CAP S+KAN R	Relapsed
PER06200941306	2009	1306	M/19	Lima	■■■■■■■■■■■■■■■■■■■■■■■■■■■■■■□■□□□□■■■□■■■	777777777720731	H3	49	253533334732243	240	CAP S+KAN R	Relapsed
PER06200843450	2008	3450	F/24	Lima	■■■■■■■■■■■■■■■■■■■■■■■■■■■■■■□■□□□□■■■□■■■	777777777720731	H3	49	253533235632243	Orph026	CAP R+KAN R	Relapsed
PER062008489	2008	89	F/39	Lima	■■■■■■■■■■■■■■■■■■■■■■■■■■■■■■□■□□□□■■■■■■■	777777777720771	H3	50	253533335612243	Orph033	CAP S+KAN R	Relapsed
PER0620084163	2008	163	M/25	Lima	■■■■■■■■■■■■■■■■■■■■■■■■■■■■■■□■□□□□■■■■■■■	777777777720771	H3	50	253533334732243	240	CAP R+KAN R	Relapsed
PER0620094817	2009	817	M/72	Lima	■■■■■■■■■■■■■■■■■■■■■■■■■■■■■■□■□□□□■■■■■■■	777777777720771	H3	50	253533334742453	Orph032	CAP R+KAN R	Relapsed
PER0620094830	2009	830	M/32	Lima	■■■■■■■■■■■■■■■■■■■■■■■■■■■■■■□■□□□□■■■■■■■	777777777720771	H3	50	263533334632243	Orph036	CAP S+KAN R	Relapsed
PER0620094924	2009	924	M/18	Lima	■■■■■■■■■■■■■■■■■■■■■■■■■■■■■■□■□□□□■■■■■■■	777777777720771	H3	50	253533335643243	Orph034	CAP R+KAN R	New
PER06200941503	2009	1503	F/43	Callao	■■■■■■■■■■■■■■■■■■■■■■■■■■■■■■□■□□□□■■■■■■■	777777777720771	H3	50	253634235632242	Orph035	CAP R+KAN R	New
PER06200941545	2009	1545	F/24	Lima	■■■■■■■■■■■■■■■■■■■■■■■■■■■■■■□■□□□□■■■■■■■	777777777720771	H3	50	233533334732343	Orph028	CAP R+KAN R	Relapsed
PER06200741678	2007	1678	M/26	Lima	■■■■■■■■■■■■■■■■■■■■■■■■■■■■■■□■□□□□■■■■■■■	777777777720771	H3	50	253533335632243	234	CAP R+KAN R	Relapsed
PER06200841877	2008	1877	M/20	Lima	■■■■■■■■■■■■■■■■■■■■■■■■■■■■■■□■□□□□■■■■■■■	777777777720771	H3	50	253533334732243	240	CAP R+KAN R	New
PER06200841906	2008	1906	M/25	Lima	■■■■■■■■■■■■■■■■■■■■■■■■■■■■■■□■□□□□■■■■■■■	777777777720771	H3	50	233753246824543	235	CAP R+KAN R	Relapsed
PER06200842129	2008	2129	M/29	Lima	■■■■■■■■■■■■■■■■■■■■■■■■■■■■■■□■□□□□■■■■■■■	777777777720771	H3	50	243534334833223	237	CAP S+KAN R	Relapsed
PER06200842377	2008	2377	M/54	Callao	■■■■■■■■■■■■■■■■■■■■■■■■■■■■■■□■□□□□■■■■■■■	777777777720771	H3	50	243534334843323	Orph030	CAP R+KAN S	Relapsed
PER06200842494	2008	2494	F/34	Tacna	■■■■■■■■■■■■■■■■■■■■■■■■■■■■■■□■□□□□■■■■■■■	777777777720771	H3	50	243533336733243	Orph029	CAP R+KAN S	Relapsed
PER06200842666	2008	2666	F/26	Lima	■■■■■■■■■■■■■■■■■■■■■■■■■■■■■■□■□□□□■■■■■■■	777777777720771	H3	50	253533335632243	234	CAP R+KAN R	Relapsed
PER06200842692	2008	2692	M/29	Lima	■■■■■■■■■■■■■■■■■■■■■■■■■■■■■■□■□□□□■■■■■■■	777777777720771	H3	50	232423-3-621332	Orph038	CAP R+KAN R	Relapsed

PER06200842700	2008	2700	M/63	Arequipa	■■■■■■■■■■■■■■■■■■■■■■■■■■■■■■□■□□□□■■■■■■■	777777777720771	H3	50	25-533335-32243	Orph041	CAP R+KAN R	Relapsed
PER06200842748	2008	2748	F/27	Lima	■■■■■■■■■■■■■■■■■■■■■■■■■■■■■■□■□□□□■■■■■■■	777777777720771	H3	50	25-4332355-2243	Orph040	CAP R+KAN R	Relapsed
PER06200842768	2008	2768	F/45	Lima	■■■■■■■■■■■■■■■■■■■■■■■■■■■■■■□■□□□□■■■■■■■	777777777720771	H3	50	23-414443222323	Orph039	CAP R+KAN R	Relapsed
PER06200843345	2008	3345	M/72	Lima	■■■■■■■■■■■■■■■■■■■■■■■■■■■■■■□■□□□□■■■■■■■	777777777720771	H3	50	253533334732243	240	CAP R+KAN R	Relapsed
PER06200843405	2008	3405	M/43	Lima	■■■■■■■■■■■■■■■■■■■■■■■■■■■■■■□■□□□□■■■■■■■	777777777720771	H3	50	253533334532243	Orph031	CAP R+KAN R	Relapsed
PER06200843487	2008	3487	M/26	Lima	■■■■■■■■■■■■■■■■■■■■■■■■■■■■■■□■□□□□■■■■■■■	777777777720771	H3	50	264543335643253	Orph037	CAP R+KAN R	Relapsed
PER06200842270	2008	2270	F/31	Lima	■■■■■■■■■■■■■■■■■■■■■■■■■■■■■■■■□□□□■■■□■■■	777777777760731	T2	52	242412242823412	Orph042	CAP S+KAN R	Relapsed
PER06200842388	2008	2388	M/72	Lima	■■■■■■■■■■■■■■■■■■■■■■■■■■■■■■■■□□□□■■■□■■■	777777777760731	T2	52	253533334732243	240	CAP S+KAN R	Relapsed
PER06200842455	2008	2455	M/42	Lima	■■■■■■■■■■■■■■■■■■■■■■■■■■■■■■■■□□□□■■■□■■■	777777777760731	T2	52	254635333833323	Orph043	CAP R+KAN R	Relapsed
PER0620094991	2009	991	M/28	Callao	■■■■■■■■■■■■■■■■■■■■■■■■■■■■■■■■□□□□■■■■■■■	777777777760771	T1	53	223526443222323	Orph045	CAP R+KAN R	Relapsed
PER06200941124	2009	1124	M/24	Lima	■■■■■■■■■■■■■■■■■■■■■■■■■■■■■■■■□□□□■■■■■■■	777777777760771	T1	53	233533334723323	Orph048	CAP R+KAN R	Relapsed
PER06200941392	2009	1392	F/21	Lima	■■■■■■■■■■■■■■■■■■■■■■■■■■■■■■■■□□□□■■■■■■■	777777777760771	T1	53	231534344532225	Orph046	CAP S+KAN R	Relapsed
PER06200842776	2008	2776	F/32	Lima	■■■■■■■■■■■■■■■■■■■■■■■■■■■■■■■■□□□□■■■■■■■	777777777760771	T1	53	242531244624312	239	CAP R+KAN R	Relapsed
PER06200842851	2008	2851	F/41	Lima	■■■■■■■■■■■■■■■■■■■■■■■■■■■■■■■■□□□□■■■■■■■	777777777760771	T1	53	222424443222323	Orph044	CAP R+KAN R	Relapsed
PER06200843364	2008	3364	F/29	Lima	■■■■■■■■■■■■■■■■■■■■■■■■■■■■■■■■□□□□■■■■■■■	777777777760771	T1	53	243534334833223	237	CAP R+KAN S	Relapsed
PER06200842436a	2008	2436a	M/38	Lima	■■■■■■■■■■■■■■■■■■■■■■■■■■■■■■■■□□□□■■■■■■■	777777777760771	T1	53	233524443222333	Orph047	CAP R+KAN R	Relapsed
PER06200941571	2009	1571	M/30	Lima	■■■□□□□□□□□□□■■■■□■■■■■■■■■■■■■■□□□□■■■■■■■	700036777760771	X3	91	253443444634313	Orph049	CAP R+KAN R	Relapsed
PER06200741803	2007	1803	F/26	Lima	■■■■■■■■■■■■□■■■■■■■□□□□■■■■■■■■□□□□■■■■■■■	777737607760771	LAM5	93	253533335652243	Orph051	CAP R+KAN R	Relapsed
PER06200842207	2008	2207	F/23	Lima	■■■■■■■■■■■■□■■■■■■■□□□□■■■■■■■■□□□□■■■■■■■	777737607760771	LAM5	93	243534333722322	Orph050	CAP S+KAN R	Relapsed
PER06200842738	2008	2738	M/17	Lima	■■■■■■■■■■■■□■■■■■■■□□□□■■■■■■■■□□□□■■■■■■■	777737607760771	LAM5	93	242512242823412	242	CAP R+KAN R	Relapsed
PER06200842754	2008	2754	F/23	Lima	■■■■■■■■■■■■■□□□□■■■■■■■■■■■■■■■□□□□■■■■■■■	777741777760771	T1	189	232423334822323	Orph052	CAP R+KAN R	Relapsed
PER062008488	2008	88	F/23	Lima	■■■■■■■■■■■■■□□□□□■■■■■■■■■■■■■■□□□□■■■■■■■	777740777760771	T1	219	243534333833323	236	CAP R+KAN R	Relapsed
PER06200941337	2009	1337	M/24	Lima	■■■■■■■■■■■■■□□□□□■■■■■■■■■■■■■■□□□□■■■■■■■	777740777760771	T1	219	243534334833223	237	CAP S+KAN R	New
PER06200741507	2007	1507	F/23	Lima	■■■■■■■■■■■■■□□□□□■■■■■■■■■■■■■■□□□□■■■■■■■	777740777760771	T1	219	243534334833323	233	CAP S+KAN R	Relapsed
PER06200741799	2007	1799	M/43	Lima	■■■■■■■■■■■■■□□□□□■■■■■■■■■■■■■■□□□□■■■■■■■	777740777760771	T1	219	242531244624312	239	CAP R+KAN R	Relapsed
PER06200842082	2008	2082	F/24	Lima	■■■■■■■■■■■■■□□□□□■■■■■■■■■■■■■■□□□□■■■■■■■	777740777760771	T1	219	222642435724332	Orph053	CAP S+KAN R	Relapsed
PER06200842348	2008	2348	F/31	Lima	■■■■■■■■■■■■■□□□□□■■■■■■■■■■■■■■□□□□■■■■■■■	777740777760771	T1	219	242512242823412	242	CAP R+KAN R	Relapsed
PER06200842365	2008	2365	M/33	Lima	■■■■■■■■■■■■■□□□□□■■■■■■■■■■■■■■□□□□■■■■■■■	777740777760771	T1	219	253534333833323	Orph061	CAP R+KAN R	Relapsed
PER06200842440	2008	2440	M/22	Lima	■■■■■■■■■■■■■□□□□□■■■■■■■■■■■■■■□□□□■■■■■■■	777740777760771	T1	219	255633352535423	Orph062	CAP R+KAN S	Relapsed
PER06200842464	2008	2464	F/34	Tacna	■■■■■■■■■■■■■□□□□□■■■■■■■■■■■■■■□□□□■■■■■■■	777740777760771	T1	219	231532344532225	Orph054	CAP R+KAN S	Relapsed
PER06200842562	2008	2562	F/21	Lima	■■■■■■■■■■■■■□□□□□■■■■■■■■■■■■■■□□□□■■■■■■■	777740777760771	T1	219	243534334833323	233	CAP R+KAN R	Relapsed
PER06200842601	2008	2601	M/27	Lima	■■■■■■■■■■■■■□□□□□■■■■■■■■■■■■■■□□□□■■■■■■■	777740777760771	T1	219	243534333833223	Orph056	CAP R+KAN S	Relapsed
PER06200842653	2008	2653	M/34	Lima	■■■■■■■■■■■■■□□□□□■■■■■■■■■■■■■■□□□□■■■■■■■	777740777760771	T1	219	252533335622243	Orph060	CAP R+KAN R	Relapsed
PER06200842875	2008	2875	M/31	Lima	■■■■■■■■■■■■■□□□□□■■■■■■■■■■■■■■□□□□■■■■■■■	777740777760771	T1	219	243544333843323	Orph059	CAP R+KAN R	Relapsed
PER06200842885	2008	2885	M/54	Callao	■■■■■■■■■■■■■□□□□□■■■■■■■■■■■■■■□□□□■■■■■■■	777740777760771	T1	219	243534334933323	Orph058	CAP R+KAN S	Relapsed
PER06200843250	2008	3250	F/23	Lima	■■■■■■■■■■■■■□□□□□■■■■■■■■■■■■■■□□□□■■■■■■■	777740777760771	T1	219	243534333833323	236	CAP R+KAN R	Relapsed
PER06200843300	2008	3300	F/19	Lima	■■■■■■■■■■■■■□□□□□■■■■■■■■■■■■■■□□□□■■■■■■■	777740777760771	T1	219	243534333833323	236	CAP R+KAN R	Relapsed
PER06200843302	2008	3302	M/16	Callao	■■■■■■■■■■■■■□□□□□■■■■■■■■■■■■■■□□□□■■■■■■■	777740777760771	T1	219	243534233833323	Orph055	CAP R+KAN R	Relapsed
PER06200843439	2008	3439	M/21	Lima	■■■■■■■■■■■■■□□□□□■■■■■■■■■■■■■■□□□□■■■■■■■	777740777760771	T1	219	243534333833323	236	CAP R+KAN S	Relapsed
PER06200843489	2008	3489	M/24	Lima	■■■■■■■■■■■■■□□□□□■■■■■■■■■■■■■■□□□□■■■■■■■	777740777760771	T1	219	241531244822312	238	CAP S+KAN R	New
PER06200741249b	2007	1249b	M/50	Lima	■■■■■■■■■■■■■□□□□□■■■■■■■■■■■■■■□□□□■■■■■■■	777740777760771	T1	219	243534333833323	236	CAP R+KAN R	New
PER06200741455a	2007	1455a	M/27	Lima	■■■■■■■■■■■■■□□□□□■■■■■■■■■■■■■■□□□□■■■■■■■	777740777760771	T1	219	243534333833322	Orph057	CAP R+KAN S	Relapsed
PER06200741823	2007	1823	M/47	Lima	■■■■■■■■■■■■■■■■□□□□□■■■■■■■□■■■□□□□■■■■■■■	777774077560771	Unknown	222	253533335632243	234	CAP R+KAN R	Relapsed
PER06200842753	2008	2753	M/32	Lima	■■■■■■■■■■■■■■■■■■■■□■■■■■■■■■■■□□□□■■■■■■■	777777677760771	T1	291	231422242523312	Orph063	CAP R+KAN R	Relapsed
PER0620094689	2009	689	F/20	Lima	□□□■■■■■■■■■■■■■■■■■□□□□■■■■■■■■□□□□■■■■■■■	077777607760771	LAM1	469	252642254833323	Orph066	CAP R+KAN R	Relapsed
PER06200843044	2008	3044	M/35	Lima	□□□■■■■■■■■■■■■■■■■■□□□□■■■■■■■■□□□□■■■■■■■	077777607760771	LAM1	469	241531244822312	238	CAP R+KAN R	Relapsed
PER06200843320	2008	3320	M/25	Madre de Dios	□□□■■■■■■■■■■■■■■■■■□□□□■■■■■■■■□□□□■■■■■■■	077777607760771	LAM1	469	243534333843333	Orph065	CAP R+KAN R	Relapsed
PER06200842862a	2008	2862a	M/32	Lima	□□□■■■■■■■■■■■■■■■■■□□□□■■■■■■■■□□□□■■■■■■■	077777607760771	LAM1	469	201531244822312	Orph064	CAP R+KAN R	Relapsed
PER06200842862b	2008	2862b	M/32	Lima	□□□■■■■■■■■■■■■■■■■■□□□□■■■■■■■■□□□□■■■■■■■	077777607760771	LAM1	469	241531244822312	238	CAP R+KAN R	Relapsed
PER0620084168	2008	168	M/49	Callao	■■■■■□■■■■■■■■■■■■■■■■■■■■■■■■■■□□□□■■■■■■■	767777777760771	T1	1122	231520242524212	Orph067	CAP S+KAN R	Relapsed
PER06200843298	2008	3298	F/17	Ica	■■■■■■■■■■■■□■■■■■□■□□□□■■■■■■■■□□□□■■■■■■■	777737207760771	LAM5	1160	242531244624312	239	CAP R+KAN R	New
PER0620094587	2009	587	M/38	Lima	■■■■■■■■■■■■■■■■■■■□□□□□■■■■□■■■□□□□■■■□■■■	777777407560731	LAM	1355	243522242412412	Orph068	CAP R+KAN R	Relapsed
PER0620094647	2009	647	M/51	Lima	■■■■■■■■■■■■■■■■■■■□□□□□■■■■□■■■□□□□■■■□■■■	777777407560731	LAM	1355	243522242523412	241	CAP R+KAN R	Relapsed
PER0620094741	2009	741	M/55	Lima	■■■■■■■■■■■■■■■■■■■□□□□□■■■■□■■■□□□□■■■□■■■	777777407560731	LAM	1355	253623242523412	Orph071	CAP R+KAN R	Relapsed
PER06200941199	2009	1199	M/55	Lima	■■■■■■■■■■■■■■■■■■■□□□□□■■■■□■■■□□□□■■■□■■■	777777407560731	LAM	1355	253522242523412	Orph070	CAP R+KAN R	Relapsed
PER06200842880	2008	2880	F/23	Lima	■■■■■■■■■■■■■■■■■■■□□□□□■■■■□■■■□□□□■■■□■■■	777777407560731	LAM	1355	244432242524422	Orph069	CAP R+KAN R	Relapsed
PER06200843184	2008	3184	M/49	Lima	■■■■■■■■■■■■■■■■■■■□□□□□■■■■□■■■□□□□■■■□■■■	777777407560731	LAM	1355	243522242523412	241	CAP R+KAN S	Relapsed
PER06200842436b	2008	2436b	M/38	Lima	■■■■■■■■■■■■■■■■■■■□□□□□■■■■□■■■□□□□■■■□■■■	777777407560731	LAM	1355	233524-43222323	Orph072	CAP R+KAN R	Relapsed
PER0620094843	2009	843	M/48	Lima	■■■■■■■■■■■■■■■■■■■■■■■■■■■■□□■■□□□□■■■■■■■	777777777460771	T1	1905	243524343222323	Orph074	CAP S+KAN R	Relapsed
PER06200843037	2008	3037	M/48	Lima	■■■■■■■■■■■■■■■■■■■■■■■■■■■■□□■■□□□□■■■■■■■	777777777460771	T1	1905	233535343222322	Orph073	CAP S+KAN R	Relapsed
PER0620084199	2008	199	M/53	Lima	■■■■■□■■■■■■■■■■■■■■■■■■■□□□□□□■□□□□■■■■■■■	767777774020771	H1	2375	253533335602223	Orph075	CAP R+KAN R	Relapsed

PER0620084270	2008	270	M/34	Lima	■■■■■□■■■■■■■■■■■■■■■■■■■□□□□□□■□□□□■■■■■■■	767777774020771	H1	2375	253534336732243	Orph076	CAP R+KAN R	Relapsed
PER06200941421	2009	1421	M/51	Lima	■■■■■■■■■■■■■■■■■■■□□□□□■■■■□■■■□□□□■■■■■■■	777777407560771	LAM6	2502	243522242523412	241	CAP R+KAN R	Relapsed
PER06200941471	2009	1471	F/34	Lima	■■■■■■■■■■■■■■■■■■■□□□□□■■■■□■■■□□□□■■■■■■■	777777407560771	LAM6	2502	243522242523412	241	CAP R+KAN R	Relapsed
PER06200842987	2008	2987	M/34	Lima	■■■■■■■■■■■■■■■■■■■■□□□□■■■■■■■■□□□□■□□■■■■	777777607760471	LAM9	2940	243520242624313	Orph077	CAP S+KAN R	Relapsed
PER0620084118	2008	118	F/70	Junin	■■■■■■■■■■■■■■■■□□□□□■■■■■■□□□□■□□□□■■■■■■■	777774077020771	H3	3001	242512242823412	242	CAP R+KAN R	Relapsed
PER0620094621	2009	621	M/35	Lima	■■■■■■■■■■■■■■■■□□□□□■■■■■■□□□□■□□□□■■■■■■■	777774077020771	H3	3001	242512242823412	242	CAP R+KAN R	Relapsed
PER06200941043	2009	1043	M/52	Lima	■■■■■■■■■■■■■■■■□□□□□■■■■■■□□□□■□□□□■■■■■■■	777774077020771	H3	3001	242513242823412	243	CAP R+KAN R	Relapsed
PER06200941525	2009	1525	M/22	Lima	■■■■■■■■■■■■■■■■□□□□□■■■■■■□□□□■□□□□■■■■■■■	777774077020771	H3	3001	242512242823412	242	CAP R+KAN R	New
PER06200941569	2009	1569	M/61	Lima	■■■■■■■■■■■■■■■■□□□□□■■■■■■□□□□■□□□□■■■■■■■	777774077020771	H3	3001	242512242923512	Orph079	CAP S+KAN R	Relapsed
PER06200941584	2009	1584	M/42	Junin	■■■■■■■■■■■■■■■■□□□□□■■■■■■□□□□■□□□□■■■■■■■	777774077020771	H3	3001	242512242823412	242	CAP S+KAN R	Relapsed
PER06200842853	2008	2853	M/42	Callao	■■■■■■■■■■■■■■■■□□□□□■■■■■■□□□□■□□□□■■■■■■■	777774077020771	H3	3001	242512242823412	242	CAP R+KAN R	Relapsed
PER06200842876	2008	2876	F/40	Lima	■■■■■■■■■■■■■■■■□□□□□■■■■■■□□□□■□□□□■■■■■■■	777774077020771	H3	3001	242512242823412	242	CAP R+KAN R	Relapsed
PER06200843011	2008	3011	M/42	Lima	■■■■■■■■■■■■■■■■□□□□□■■■■■■□□□□■□□□□■■■■■■■	777774077020771	H3	3001	242512242823413	Orph078	CAP R+KAN R	Relapsed
PER06200843325	2008	3325	M/22	Lima	■■■■■■■■■■■■■■■■□□□□□■■■■■■□□□□■□□□□■■■■■■■	777774077020771	H3	3001	242522242823412	Orph080	CAP R+KAN R	New
PER06200741455b	2007	1455b	M/27	Lima	■■■■■■■■■■■■■■■■□□□□□■■■■■■□□□□■□□□□■■■■■■■	777774077020771	H3	3001	242532242823412	Orph081	CAP R+KAN S	Relapsed
PER06200842750	2008	2750	M/26	Lima	■■■■■■■■■■■■■■■■□■□□□■■■■■■■□■■■□□□□■■■■■■■	777775077560771	T1	3777*	242422242413312	Orph082	CAP R+KAN R	Relapsed
PER06200842027	2008	2027	M/40	Callao	■■■■■■■■■■■■■■■■□■□□□■■■■■■□□□□■□□□□■■■■■■■	777775077020771	H3	3778*	242513242823412	243	CAP R+KAN R	Relapsed
PER06200842080	2008	2080	F/27	Lima	■■■■■■■■■■■■■■■■□■□□□■■■■■■□□□□■□□□□■■■■■■■	777775077020771	H3	3778*	253523352824422	Orph085	CAP R+KAN R	Relapsed
PER06200842302	2008	2302	M/16	Lima	■■■■■■■■■■■■■■■■□■□□□■■■■■■□□□□■□□□□■■■■■■■	777775077020771	H3	3778*	242531244624322	Orph083	CAP R+KAN S	Relapsed
PER06200842369	2008	2369	M/38	Lima	■■■■■■■■■■■■■■■■□■□□□■■■■■■□□□□■□□□□■■■■■■■	777775077020771	H3	3778*	244635553223333	Orph084	CAP R+KAN R	Relapsed
PER06200842482	2008	2482	M/24	Lima	■■■■■■■■■■■■■■■■□■□□□■■■■■■□□□□■□□□□■■■■■■■	777775077020771	H3	3778*	253534334844323	Orph086	CAP S+KAN R	New
PER0620084240	2008	240	M/45	Junin	■■■■■□■■■■■■■■■■□□□□□■■■■■■□□□□■□□□□■■■■■■■	767774077020771	H3	3779*	242512242822412	Orph087	CAP R+KAN R	Relapsed
PER0620094302	2009	302	M/40	Callao	■■■■■□■■■■■■■■■■□□□□□■■■■■■□□□□■□□□□■■■■■■■	767774077020771	H3	3779*	242512242823412	242	CAP R+KAN R	Relapsed
PER0620084164	2008	164	F/53	Lima	■■■□□□□□□□□□□■■■■□■■■■■■■■■■■■■■□□□□□■■■■■■■	700036777740771	X3	3780*	243331335332343	Orph088	CAP R+KAN R	Relapsed
PER06200741698	2007	1698	F/31	Lima	■■■■□□■■■■■■■■■■□□□□□■■■■■■□□□□■□□□□■■■■■■■	747774077020771	H3	Orphan1	232512242723412	Orph089	CAP R+KAN R	Relapsed
PER06200741714	2007	1714	M/31	Callao	■■■■□■■■■■■■■□□□□□■■■■■■■■■■■■■■□□□□■■■■■■■	757740777760771	T1	Orphan2	232534333732323	Orph091	CAP R+KAN R	Relapsed
PER06200842770	2008	2770	M/27	Lima	■■■■■■■■■■■■■■■□□■□□□□□□□□□□□□□□□□□□□□□■■■■	777771000000071	Unknown	Orphan3	223533335-12243	Orph093	CAP S+KAN R	Relapsed
PER0620094412	2009	412	M/33	Lima	■■■■■□□■■■■■■□□□□□■■■■■■■■■■■■■■□□□□■■■■■■■	763740777760771	T1	Orphan4	253544333833323	Orph092	CAP R+KAN R	Relapsed
PER0620094531	2009	531	M/28	Callao	■■■■■□■■■■■■□■■■■■■■■■■■■■■■■■■□□□□■■■■■■■	767737777760771	T3	Orphan5	232516443222423	Orph090	CAP R+KAN R	Relapsed

Unique strains matching a preexisting pattern in the SITVIT2 database are classified as SITs, whereas in case of no match, they are designated as ‘‘orphan", and highlighted in blue.

*SITs followed by an asterisk and highlighted in yellow indicate "newly created shared-type" after match with another orphan in the database, or due to 2 or more strains belonging to a new pattern within this study.
