# Supplementary material for: Characterization of the Genetic Diversity of Extensively-Drug Resistant Mycobacterium tuberculosis Clinical Isolates from Pulmonary Tuberculosis Patients in Peru
Source: PLoS One. 2014 Dec 9;9(12):e112789. doi: 10.1371/journal.pone.0112789 (PMC4260790; doi:10.1371/journal.pone.0112789)
Supplement: S3 Table — A comparison of the proportion of all SITs found in this study as compared to the other strains isolated in Peru and neighboring regions (Northern America, Southern America, Central America and Caribbean), recorded in the SITVIT2 database. (PDF) [file pone.0112789.s007.pdf]

<u>Supplemental Table S3:</u> A comparison of the proportion of all SITs found in this study as compared to the other strains isolated in Peru and neighbouring regions (Northern America, Southern America, Central America and Caribbean), recorded in the SITVIT2 database.

SIT	Spoligotype Description	Octal value	This study n/t (%)	Distribution In Neighboring Regions			
				Peru (Without Study) n/t (%)	AMER-N n/t (%)	AMER-C & CARI n/t (%)	AMER-S (without study) n/t (%)
1	□□□□□□□□□□□□□□□□□□□□□□□□□□□□□□□□□□■■■■■■■■■	000000000003771	13/142 (9,15)	50/886 (5.64)	1994/14557 (13.70)	65/3197 (2.03)	91/9716 (0.94)
42	■■■■■■■■■■■■■■■■■■■■□□□□■■■■■■■■□□□□■■■■■■■	777777607760771	4/142 (2,82)	69/886 (7.79)	397/14557 (2.73)	206/3197 (6.44)	973/9716 (10.01)
47	■■■■■■■■■■■■■■■■■■■■■■■■■□□□□□□■□□□□■■■■■■■	777777774020771	16/142 (11,27)	32/886 (3.61)	243/14557 (1.67)	58/3197 (1.81)	177/9716 (1.82)
49	■■■■■■■■■■■■■■■■■■■■■■■■■■■■■■□■□□□□■■■□■■■	777777777720731	4/142 (2,82)	9/886 (1.02)	26/14557 (0.18)	9/3197 (0.28)	19/9716 (0.20)
50	■■■■■■■■■■■■■■■■■■■■■■■■■■■■■■□■□□□□■■■■■■■	777777777720771	21/142 (14,79)	139/886 (15.69)	595/14557 (4.09)	160/3197 (5.00)	579/9716 (5.96)
52	■■■■■■■■■■■■■■■■■■■■■■■■■■■■■■■■□□□□■■■□■■■	777777777760731	3/142 (2,11)	1/886 (0.11)	111/14557 (0.76)	41/3197 (1.28)	18/9716 (0.19)
53	■■■■■■■■■■■■■■■■■■■■■■■■■■■■■■■■□□□□■■■■■■■	777777777760771	7/142 (4,93)	111/886 (12.53)	847/14557 (5.82)	359/3197 (11.23)	749/9716 (7.71)
91	■■■□□□□□□□□□□■■■■□■■■■■■■■■■■■■■■□□□□■■■■■■■	700036777760771	1/142 (0,7)	24/886 (2.71)	126/14557 (0.87)	37/3197 (1.16)	55/9716 (0.57)
93	■■■■■■■■■■■■□■■■■■■■□□□□■■■■■■■■□□□□■■■■■■■	777737607760771	3/142 (2,11)	14/886 (1.58)	75/14557 (0.52)	43/3197 (1.35)	170/9716 (1.75)
189	■■■■■■■■■■■■■□□□□■■■■■■■■■■■■■■■□□□□■■■■■■■	777741777760771	1/142 (0,7)	1/886 (0.11)	3/14557 (0.02)	2/3197 (0.06)	1/9716 (0.01)
219	■■■■■■■■■■■■■□□□□□■■■■■■■■■■■■■■□□□□■■■■■■■	777740777760771	21/142 (14,79)	7/886 (0.79)	31/14557 (0.21)	0/3197 (0.00)	7/9716 (0.07)
222	■■■■■■■■■■■■■■■■□□□□□■■■■■■■□■■■□□□□■■■■■■■	777774077560771	1/142 (0,7)	26/886 (2.93)	17/14557 (0.12)	1/3197 (0.03)	27/9716 (0.28)
291	■■■■■■■■■■■■■■■■■■■■□■■■■■■■■■■■□□□□■■■■■■■	777777677760771	1/142 (0,7)	1/886 (0.11)	1/14557 (0.01)	5/3197 (0.16)	23/9716 (0.24)
469	□□□■■■■■■■■■■■■■■■■■□□□□■■■■■■■■□□□□■■■■■■■	077777607760771	5/142 (3,52)	4/886 (0.45)	13/14557 (0.09)	2/3197 (0.06)	6/9716 (0.06)
1122	■■■■■□■■■■■■■■■■■■■■■■■■■■■■■■■■□□□□■■■■■■■	767777777760771	1/142 (0,7)	1/886 (0.11)	4/14557 (0.03)	0/3197 (0.00)	2/9716 (0.02)
1160	■■■■■■■■■■■■□■■■■■□■□□□□■■■■■■■■□□□□■■■■■■■	777737207760771	1/142 (0,7)	0/886 (0.00)	2/14557 (0.01)	0/3197 (0.00)	1/9716 (0.01)
1355	■■■■■■■■■■■■■■■■■■■□□□□□■■■■□■■■□□□□■■■□■■■	777777407560731	7/142 (4,93)	26/886 (2.93)	7/14557 (0.05)	0/3197 (0.00)	27/9716 (0.28)
1905	■■■■■■■■■■■■■■■■■■■■■■■■■■■■□□■■□□□□■■■■■■■	777777777460771	2/142 (1,41)	0/886 (0.00)	0/14557 (0.00)	0/3197 (0.00)	18/9716 (0.19)
2375	■■■■■□■■■■■■■■■■■■■■■■■■■□□□□□□■□□□□■■■■■■■	767777774020771	2/142 (1,41)	0/886 (0.00)	0/14557 (0.00)	0/3197 (0.00)	0/9716 (0.00)
2502	■■■■■■■■■■■■■■■■■■■□□□□□■■■■□■■■□□□□■■■■■■■	777777407560771	2/142 (1,41)	0/886 (0.00)	0/14557 (0.00)	0/3197 (0.00)	3/9716 (0.03)
2940	■■■■■■■■■■■■■■■■■■■■□□□□■■■■■■■■□□□□■□□■■■■	777777607760471	1/142 (0,7)	0/886 (0.00)	0/14557 (0.00)	0/3197 (0.00)	5/9716 (0.05)
3001	■■■■■■■■■■■■■■■■□□□□□■■■■■■□□□□■□□□□■■■■■■■	777774077020771	11/142 (7,75)	8/886 (0.90)	1/14557 (0.01)	0/3197 (0.00)	8/9716 (0.08)
3777	■■■■■■■■■■■■■■■■□■□□□■■■■■■■□■■■□□□□■■■■■■■	777775077560771	1/142 (0,7)	0/886 (0.00)	0/14557 (0.00)	0/3197 (0.00)	0/9716 (0.00)
3778	■■■■■■■■■■■■■■■■□■□□□■■■■■■□□□□■□□□□■■■■■■■	777775077020771	5/142 (3,52)	0/886 (0.00)	0/14557 (0.00)	0/3197 (0.00)	0/9716 (0.00)
3779	■■■■■□■■■■■■■■■■□□□□□■■■■■■□□□□■□□□□■■■■■■■	767774077020771	2/142 (1,41)	0/886 (0.00)	0/14557 (0.00)	0/3197 (0.00)	0/9716 (0.00)
3780	■■■□□□□□□□□□□■■■■□■■■■■■■■■■■■■□□□□□■■■■■■■	700036777740771	1/142 (0,7)	1/886 (0.11)	0/14557 (0.00)	0/3197 (0.00)	1/9716 (0.01)
	**TOTAL**		**137/142 (96.48)**	**524/886 (56.14)**	**4493/14557 (30.86)**	**988/3197 (30.90)**	**2960/9716 (30.47)**
